# Emotion knowledge is structured in a grid-like coordinate system in the human ventromedial prefrontal cortex

**DOI:** 10.1371/journal.pbio.3003795

**Published:** 2026-11-02

**Authors:** Yumeng Ma, Philip A. Kragel

**Affiliations:** 1 Department of Psychology, Emory University, Atlanta, Georgia, United States of America; 2 Department of Psychiatry and Behavioral Sciences, Emory University, Atlanta, Georgia, United States of America; Universitat Jaume 1, SPAIN

## Abstract

Human emotional experiences vary widely in everyday life, yet they are systematically organized within a continuous affective space defined by valence and arousal. Although this circumplex organization has shaped decades of research, its neural implementation remains unclear. Here, we asked whether abstract emotion concepts are encoded using grid-like coordinate principles in the human brain. During fMRI, participants tracked trajectories through affective space conveyed through morphs of facial expressions and sequences of phrases. We found that ventromedial prefrontal cortex (vmPFC) encoded affective space with robust hexadirectional modulation characteristic of grid-like coding. This coordinate representation generalized across stimulus modalities, indicating abstraction beyond perceptual features. The grid-like signal reflected a metric organization of emotion concepts independent of overt behavior, whereas distance-related signals in vmPFC predicted trial-by-trial affective judgements. Together, these findings suggest that emotion knowledge is structured by a grid-like coordinate system in the human brain that can support predictive inference and flexible decision-making.

## Abstract 2

Human emotions are organized within a continuous affective space defined by valence and arousal, but how this conceptual structure is represented in the brain remains unclear. This study shows that the ventromedial prefrontal cortex encodes emotion concepts using a grid-like coordinate system that generalizes across modalities and supports affective judgments.

## Introduction

Humans navigate a wide range of events in everyday life, from pleasant, calm conversations to tense, conflict-laden encounters. These episodes are complex, varying in the external situations, internal bodily sensations, and potential actions that define them. To behave adaptively in such environments, the brain is thought to organize accumulated knowledge into structured representations that support prediction, inference, and flexible behavior [1,2]. Converging evidence suggests that diverse forms of knowledge, including that of physical space [3], visual objects [4], people [5], odors [6], choice options [7,8], and action plans [9], are encoded in a map-like way in medial temporal and prefrontal systems. Such representations embed specific experiences within continuous spaces, allowing relationships among states to be expressed geometrically, enabling generalization beyond immediately available sensory inputs [2].

Among the many domains of knowledge that guide flexible behavior, information about emotional events is particularly important for survival and well-being [10]. Decades of behavioral research indicate that individuals organize emotion knowledge in a two-dimensional affective space defined by valence and arousal [11,12]. This space is consistently observed across judgments of facial [13], vocal expressions [14], emotion words [11,15], and self-reported feelings [16], suggesting that individuals abstract meaning from diverse sensory experiences to represent a shared structure of affect. This configuration, known as the affective circumplex, has been proposed to reflect the cognitive structure laypeople use to organize knowledge about emotions and guide behavior [11].

Despite the robustness of circumplex structure in behavioral data, its neural basis remains unclear. Contemporary theories explaining the brain basis of emotion have proposed that the default network constructs predictive models of the world to anticipate the needs of an organism in context [17,18]. Within this network, ventromedial prefrontal cortex (vmPFC) has been implicated in integrating long-term memory with information about the self and current goals to generate value-based predictions that guide decision-making [19,20]. These accounts explain how affective meaning can be constructed flexibly across situations, yet they do not specify the representational format used to organize emotion knowledge. As a result, they do not explain why circumplex structure emerges so consistently across modalities, tasks, and individuals [12].

One possibility is that hippocampal-prefrontal systems represent emotion knowledge in a map-like way [21]. In this account, the hippocampus represents specific emotion concepts, whereas the vmPFC and the entorhinal cortex represent the relational structure that organizes these concepts, enabling flexible generalization across contexts. However, relational structure alone does not specify the format of a map [22]. It remains unclear whether affective space is implemented in a coordinate-based metric system. In spatial and conceptual domains, such a metric structure is supported by grid-like codes. When participants navigate physical and abstract spaces, fMRI signals in vmPFC and entorhinal cortex exhibit hexadirectional modulation—a 6-fold periodic pattern aligned to movement in space [23]. Similar grid-like coding has been observed in vmPFC during economic decision-making involving an abstract value space [24]. Moreover, vmPFC activity in response to emotional films can be predicted from regularities in emotion transitions consistent with structural generalization [25]. However, direct evidence that affective space itself is represented using grid-like codes remains lacking.

To test whether affective space is implemented using a grid-like basis, here we conducted an fMRI experiment in which participants (*n* = 18) viewed emotion-laden stimuli and judged how they varied along dimensions of valence and arousal. During scanning, participants were presented with morphs of prototypical facial expressions and sequences of phrases (Fig 1A). Each trial consisted of a single trajectory defined by start- and end points in affective space. We created stimuli to provide broad coverage across the affective space and validated them via post-scan ratings of valence, arousal, and emotion categories (Fig 1B). On each trial during scanning, participants indicated whether the valence or arousal of the stimulus increased, decreased, or stayed the same. We varied the probed dimension randomly, requiring participants to track changes in both valence and arousal throughout the task.

**Fig 1:**
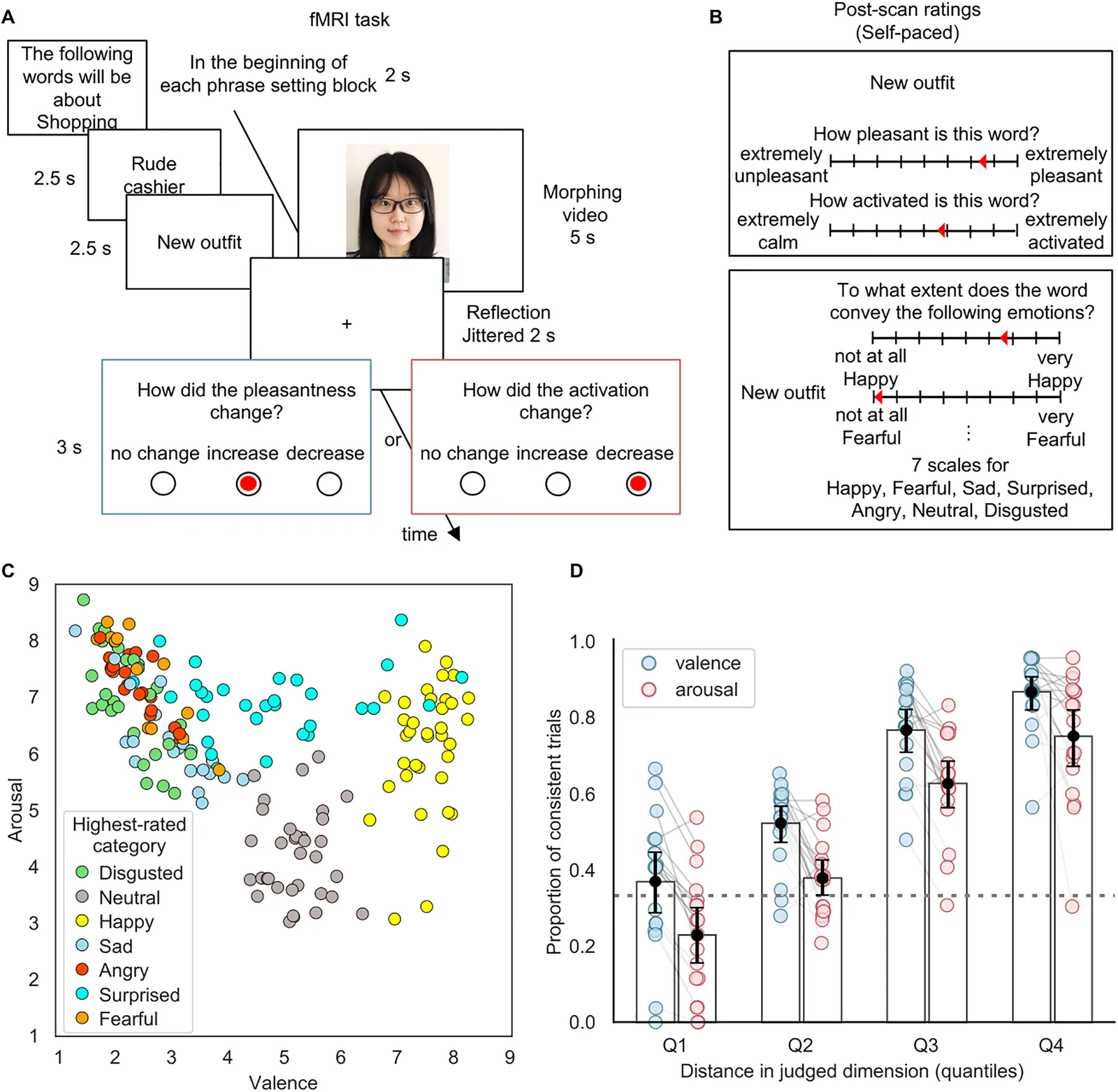
Experimental paradigm and behavior. **(A)** Schematic of a single trial in the two versions of the task (phrase and face). Each trial began with a sequence of two phrases from the same setting, or a morph between two facial expressions of the same identity. After a jittered fixation period, participants had 3 s to indicate whether valence or arousal had increased, decreased, or stayed the same. The probed dimension was split evenly across trials and revealed only on the response screen, so participants could not anticipate which dimension would be queried while viewing the stimuli. The face image shown is an illustrative example similar to the experimental stimulus; the actual stimuli were drawn from the Karolinska Directed Emotional Faces database [26]. (**B)** Rating screens for one example phrase. After scanning, participants rated each phrase and facial expression on valence and arousal, followed by ratings of seven emotion categories, using continuous scales. (**C)** Group-average valence-arousal ratings. Each point represents one stimulus and is colored according to the emotion category with the highest rating. (**D)** Proportion of trials in which in-scanner judgments were consistent with the group-average post-scan ratings across four quantiles of absolute distance in the judged dimension for both valence (blue) and arousal (pink) comparisons. The dotted line indicates chance agreement. Points represent individual participants (*n* = 18 independent participants), and points from the same participant are connected by gray lines. Bars show group means with 95% confidence intervals. Numerical data are provided in S1 Data.

By combining two perceptually distinct stimulus modalities with diverse affective trajectories, this design allowed us to test whether abstract emotion concepts are organized using a modality-invariant coordinate system. If this is the case, we would expect two outcomes. First, post-scan ratings should recover the geometric organization of affect that defines the circumplex. Second, BOLD signals in vmPFC should exhibit hexadirectional modulation as a function of trajectory direction through that space, reflecting grid-like coding that generalizes across stimulus modalities and is independent of task demands.

### Grid-like coding of affective space in vmPFC

A map-like organization of affect entails that judgments of emotional stimuli are based on a Euclidean space in which emotion concepts are embedded [11]. Indeed, analyses of the post-scan ratings revealed a two-dimensional affective structure broadly similar across participants (S1–S3 Figs; S1 and S2 Tables), stimulus modalities (S4 Fig), and rating types, including direct ratings of valence and arousal (Fig 1C) as well as multidimensional scaling (MDS) of emotion category ratings (S5 Fig). Across these complementary measures, the dominant structure in our data resembled a circumplex, with positive categories (e.g., happy, surprise) clustering toward high valence, negative categories (e.g., fear, sadness, anger, disgust) toward low valence regions, and arousal increasing from calm to highly activating categories like surprise and fear. Because the MDS solution was derived independently of any prespecified valence-arousal axes, its convergence with the geometry recovered from direct valence-arousal ratings (Procrustes disparity = 0.20, permutation *p* < .0001) provides evidence for an integrated representation of the two affective dimensions.

To examine whether this structure aligned with choice behavior during the fMRI scan, we assessed the relationship between in-scanner judgments of valence and arousal change and the affective structure derived from post-scan ratings. We found that as the distance between stimuli in the judged dimension increased, so did the agreement between individual choices during scanning and the group-average post-scan ratings (logistic regression: *β* = 0.9137, SE = 0.1212, *z* = 7.540, 95% CI [0.6770, 1.1599], *p* < .0001; overall proportion of consistent choices: range = 50.96%–66.35%; median = 56.97%, IQR = 7.21%, chance = 33.33%; balanced consistency across increase, decrease, and no-change trials: median = 57.92%, IQR = 7.05%; Fig 1D). Such a relationship was not observed for distance along the non-judged dimension (*β* = −0.0560, SE = 0.0443, *z* = −1.264, *p* = .2063), nor was there an interaction between distances along the judged and non-judged dimensions (*β* = −0.0215, SE = 0.0510, *z* = −0.421, *p* = .6737). These behavioral results indicate that participants could reliably read out task-relevant changes along the valence and arousal dimensions during scanning in a manner consistent with the group-average post-scan ratings, validating the use of rating-derived coordinates for subsequent tests of grid-like structure in the fMRI data.

When participants viewed face morphs and phrase sequences, transitions between emotion concepts could be represented as movement through affective space. If the basis for representing this space is implemented in grid-like population codes, trajectories aligned with a common grid orientation should evoke similar fMRI patterns separated by 60° rotational symmetry. To test this prediction, we estimated putative grid orientation (S3 Table) and quantified hexadirectional modulation using a cross-validated approach in which orientation was estimated and tested on independent data from two pairs of functional runs (see Materials and methods). Examining signals in a region of vmPFC previously identified as exhibiting 6-fold symmetry [4], we distinguished trials with trajectories aligned with the estimated grid axes and computed multivoxel pattern similarity across trial pairs (Fig 2B and 2C).

**Fig 2:**
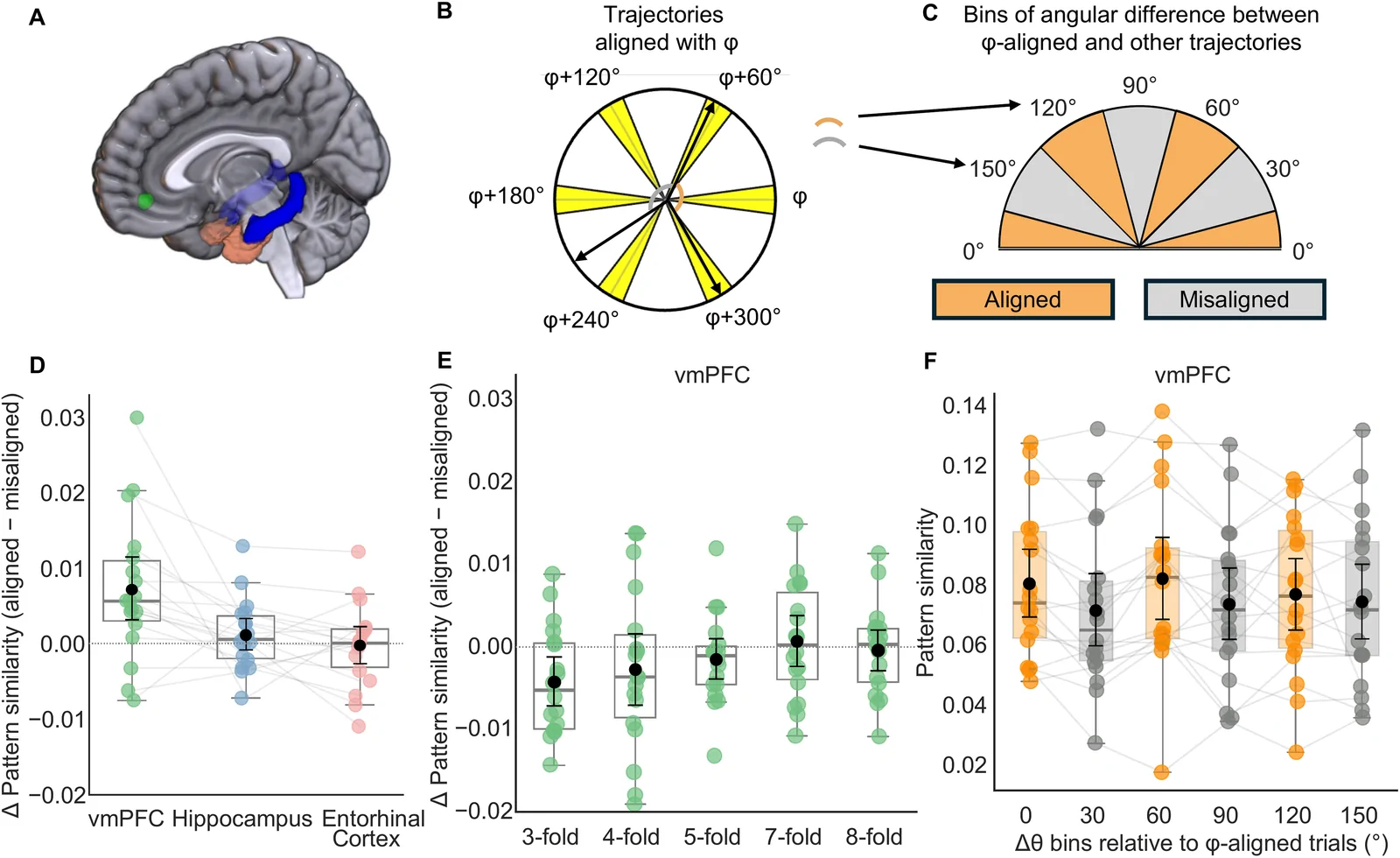
Grid-like coding of affective space in ventromedial prefrontal cortex. **(A)** Masks for regions of interest: ventromedial prefrontal cortex (vmPFC; green), hippocampus (blue), and entorhinal cortex (pink). (**B)** Trajectories with angles within ± 7.5° to the estimated grid orientation *φ* (mod 60°) were defined as *φ*-aligned (yellow). (**C)** Trials were classified as aligned (within 0° ± 15°, mod 60°; orange) or misaligned (within 30° ± 15°, mod 60°; gray) to the *φ*-aligned trials. Two example trial pairs were shown for the 120° and 150° bins (see panel **B)**. (**D)** Pattern similarity differences between aligned-aligned and aligned-misaligned trial pairs across regions. (**E)** Effects for control periodicities in vmPFC (3-fold: Δ*r* = −0.0043, 95% CI [−0.0072, −0.0012], *d* = −0.6713, *p* = .9910; 4-fold: Δ*r* = −0.0028, 95% CI [−0.0071, 0.0017], *d* = −0.2892, *p* = .8735; 5-fold: Δ*r* = −0.0015, 95% CI [−0.0039, 0.0009], *d* = −0.2927, *p* = .8700; 7-fold: Δ*r* = 0.0007, 95% CI [−0.0024, 0.0038], *d* = 0.0966, *p* = .3382; 8-fold: Δ*r* = −0.0004, 95% CI [−0.0029, 0.0021], *d* = −0.0826, *p* = .6290). (**F)** Pattern similarity as a function of angular bin (colors as in C) in vmPFC. Boxplots show the median and interquartile range; whiskers extend to 1.5× the interquartile range. Black circles and error bars denote the mean and 95% confidence interval. Each point represents one participant (*n* = 18 independent participants) and points from the same participants are connected by gray lines. A dotted line marks zero (no aligned-misaligned difference) in panels D and E. vmPFC, ventromedial prefrontal cortex. All numerical data are provided in S2 Data.

Consistent with grid-like coding, response patterns in portions of vmPFC (Fig 2A) were more similar for trajectory pairs aligned with the same 60° periodic grid orientation (*r* = .0801, SD = .0262) than for those offset by 30° (*r* = .0729, SD = .0262; Δ*r* = 0.0072, 95% CI [0.0031, 0.0115], *d* = 0.7820, *p* = .0014; Figs 2D, 2F and S6 Fig). This effect was specific to 6-fold periodicity characteristic of grid-like coding (6-fold versus other folds: Δ*r* = 0.0088, 95% CI [0.0046, 0.0133], *d* = 0.9223, *p* = .0005; Fig 2E). Simulations showed the magnitude of the pattern similarities observed for the 6-fold effect corresponds to a signal-to-noise ratio of 0.1535 (S7 Fig). We did not observe grid-like responses in the entorhinal cortex (Δ*r* = −0.0002, 95% CI [−0.0027, 0.0022], *d* = −0.0452, *p* = .5594; Fig 2D). Given the established evidence for grid cells and grid-like fMRI signals in this region across spatial and abstract domains [3,4,27], this result was unexpected. However, temporal signal-to-noise and mean signal in this region were lower (S8 Fig), which may have limited sensitivity to detect subtle effects.

As a point of comparison, we compared the modulation of response patterns in the vmPFC to those in the hippocampus, which has been found to exhibit a map-like structure [25], but not grid-like coding. Consistent with our account that the hippocampus represents specific concepts embedded within affective space, rather than the low-dimensional grid-like codes as expressed in vmPFC [21], we observed no comparable grid-like coding effect in the hippocampus (Δ*r* = 0.0011, 95% CI [−0.0008, 0.0034], *d* = 0.2498, *p* = .1568; Δ*r*_hippocampus –_ Δ*r*_vmPFC_ = −0.0060, 95% CI [−0.0105, −0.0021], *d* = −0.6592, *p* = .0042; Fig 2D).

Having established that a grid-like code of affective space was present in vmPFC, we next conducted a series of tests to assess the sensitivity of this effect to our analytical procedures. Because the number of trial pairs contributing to the aligned and misaligned conditions differed, with an average of 1,514.2 aligned trial pairs (SD = 234.1) and 1,363.8 misaligned pairs (SD = 97.2) per cross-validation fold across participants, we performed a subsampling analysis to match the number of pairs between conditions. Averaged across 10,000 subsampling iterations, the 6-fold modulation remained unchanged (Δ*r* = 0.0072, 95% CI [0.0032, 0.0115], *d* = 0.7827, *p* = .0014). Assessing other aspects of the analysis, we observed evidence of 6-fold modulation at multiple local spatial scales (S9 Fig) and localizations of vmPFC from past studies [4,6] (S4 Table). Exploratory searchlight and univariate mapping revealed effects throughout the vmPFC and other regions that have been found to exhibit 6-fold modulation in prior studies, including multiple regions of the default network (S10–S12 Figs and S5 Table).

To account for potential confounds that could increase pattern similarity between aligned trials, including affective features, emotion category, trajectory distances, face identity, phrase setting, and behavioral choice (S13–S15 Figs), we conducted a control analysis incorporating these variables as nuisance regressors. The 6-fold modulation in vmPFC remained robust in this analysis (Δ*r* = 0.0070, 95% CI [0.0033, 0.0109], *d* = 0.8416, *p* = .0008; Fig 3B). Because aligned pairs more often shared the same start category, end category, and stimulus identity or setting (S15 Fig), we also repeated the analysis after directly matching aligned and misaligned pairs on these factors. The 6-fold effect was again present in the matched subsample (Δ*r* = 0.0062, 95% CI [0.0022, 0.0106], *d* = 0.6818, *p* = 0.0026; Fig 3B).

**Fig 3:**
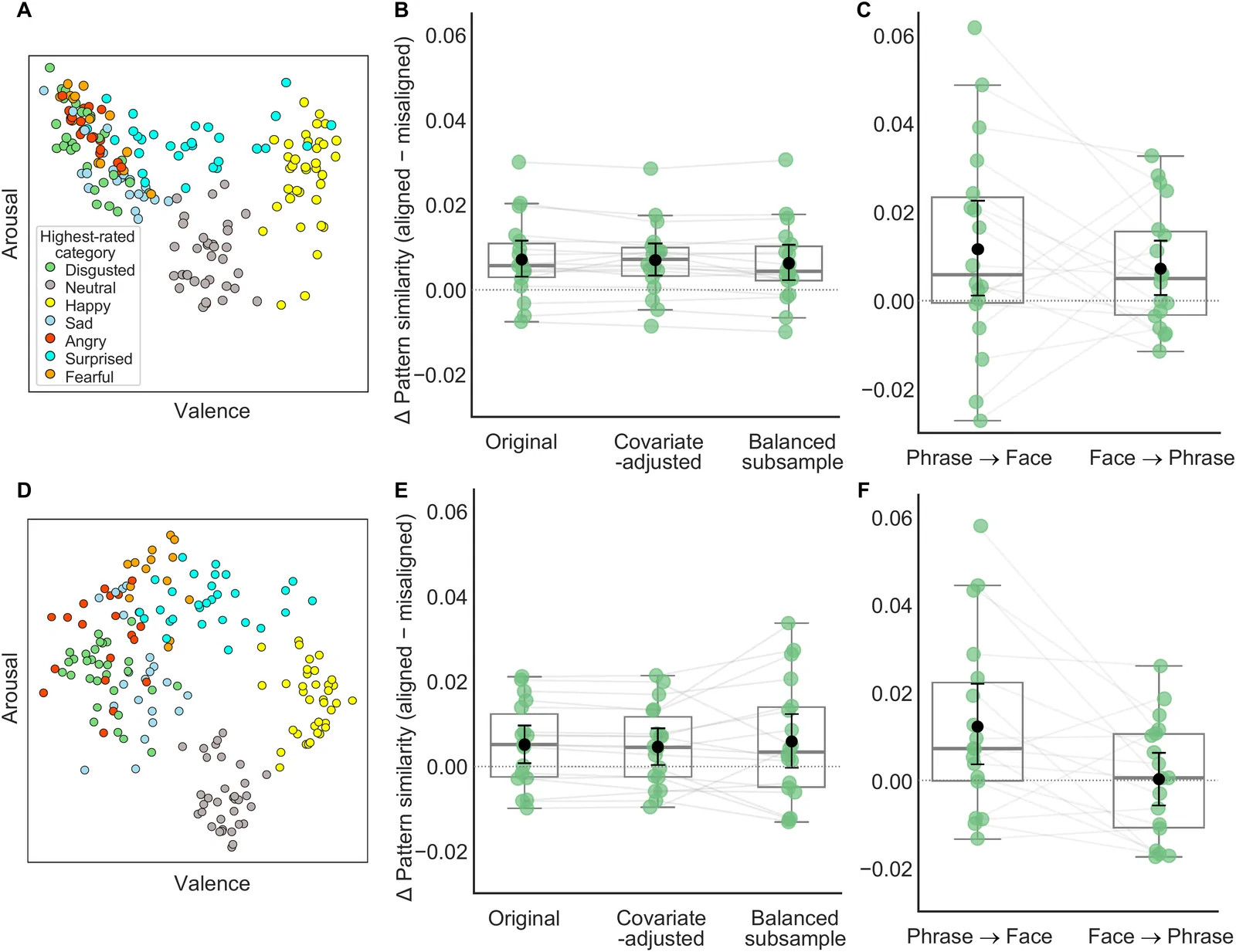
Grid-like coding of affective space in ventromedial prefrontal cortex is robust to stimulus-related confounds and analytical degrees of freedom. **(A)** Group-average valence-arousal ratings (same as Fig 1C). **(B)** Pattern similarity differences between aligned-aligned and aligned-misaligned trial pairs computed based on the space defined by valence-arousal ratings. Results are shown after controlling for potential confounding factors (covariate-adjusted), and after subsampling aligned and misaligned trial pairs to match the proportions sharing the same start category, end category, face identity or phrase setting, and behavioral choice (balanced subsample). **(C)** Pattern similarity differences between aligned-aligned and aligned-misaligned trial pairs in one modality using the grid orientation estimated from the other modality, based on space defined by valence-arousal ratings. **(D)** Two-dimensional multidimensional scaling (MDS) solution aligned to the valence-arousal space in panel A using Procrustes analysis. **(E, F)** Pattern similarity differences based on the MDS space. Boxplots show the median and interquartile range; whiskers extend to 1.5× the interquartile range. Black circles and error bars denote the mean and 95% confidence interval. Each point represents one participant (*n* = 18 independent participants) and points from the same participants are connected by gray lines. A dotted line marks zero (no aligned-misaligned difference) in panels B, C, E, and **F.** All numerical data are provided in S3 Data.

To test the robustness of the hexadirectional effect in vmPFC to alternative estimates of affective space, we repeated the analysis using trajectory angles derived from multidimensional scaling of emotion category ratings (Fig 3D). The grid-like effect in vmPFC was replicated using this alternative geometry (Δ*r* = 0.0051, 95% CI [0.0008, 0.0096], *d* = 0.5359, *p* = .0203; 6-fold versus other folds: Δ*r* = 0.0049, 95% CI [0.0006, 0.0094], *d* = 0.5091, *p* = .0245), and remained evident after controlling for potential confounds (Δ*r* = 0.0046, 95% CI [0.0004, 0.0090], *d* = 0.4935, *p* = .0288) and matched subsampling (Δ*r* = 0.0058, 95% CI [−0.0003, 0.0124], *d* = 0.4239, *p* = .0476; Fig 3E). Notably, hexadirectional modulation was present in vmPFC when affective space was defined directly from self-reported valence and arousal and when it was estimated using data-driven multidimensional scaling, indicating that grid-like representation in vmPFC is robust to independent methods of defining affective space.

### Generalization of grid-like codes across facial and linguistic stimuli

Dimensional theories of affect propose that a common cognitive structure underlies judgements about the meaning of words, facial expressions, and subjective experience more generally [11]. If vmPFC implements a grid-like basis for affective space, its orientation should generalize across stimuli that differ in perceptual features but have similar meaning and underlying geometry. Estimating grid orientation from behavioral ratings of phrase stimuli and testing on faces revealed robust 6-fold modulation in vmPFC (Phrase →Face: Δ*r* = 0.0117, 95% CI [0.0013, 0.0224], *d* = 0.5061, *p* = .0234). The reciprocal analysis produced comparable results (Face →Phrase: Δ*r* = 0.0072, 95% CI [0.0013, 0.0135], *d* = 0.5495, *p* = .0196; Fig 3C). When affective space was defined using MDS of category ratings, cross-modal generalization was observed in the phrase-to-face direction (Δ*r* = 0.0123, 95% CI [0.0038, 0.0218], *d* = 0.6242, *p* = .0096), but not from faces to phrases (Δ*r* = 0.0002, 95% CI [−0.0057, 0.0063], *d* = 0.0187, *p* = .4693; Fig 3F). These results suggest that hexadirectional coding in vmPFC may reflect a modality-invariant representation of affective space, although cross-modal generalization was not consistently observed across alternative definitions of the space.

### Grid-like coding of affective space in vmPFC is distinct from decision-related signals

The ventromedial prefrontal cortex is widely implicated in encoding decision-related variables [28], such as the subjective value of an action [29–31] emotional value [32], and decision confidence [33,34]. To determine whether the observed grid-like signals in vmPFC are linked to decision processes used to evaluate stimuli, we examined how grid-like signals relate to the consistency of judgments made during and after scanning. If the hexagonal modulation we observed resulted from a well-established map of emotion concepts, then its expression should be independent of behavioral choice. Alternatively, if the hexagonal modulation resulted from signals more closely tied to task performance (e.g., a mapping of task sets or action values), then hexagonal modulation should be more closely related to behavioral performance. Comparisons of hexadirectional modulation on trials in which judgements were consistent with consensus ratings and those that were inconsistent revealed minimal differences (Δ*r*_consistent_ − Δ*r*_inconsistent_ = 0.0049, 95% CI [−0.0031, 0.0126], *d* = 0.2880, *p* = .1220; BF10 = 0.4477; Fig 4B). This finding suggests that the strength of the grid-like signal we observed was not closely tied to choice variables in the task.

**Fig 4:**
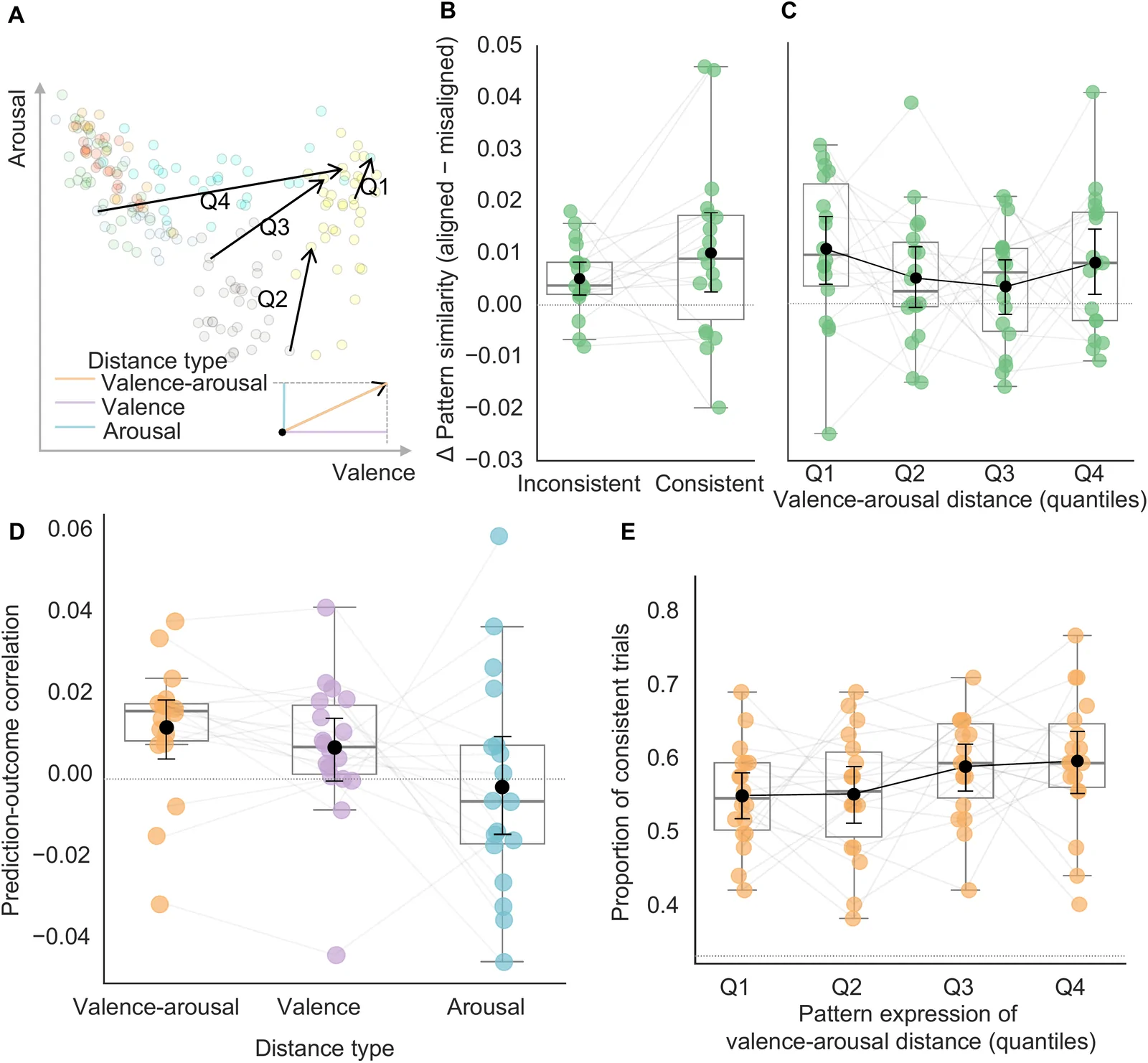
Hexadirectional modulation in vmPFC is distinct from decision-related signals. **(A)** Schematic illustration of distance measures in affective space. The background scatter plot shows group-average valence and arousal ratings for all stimuli, as shown in Fig 1C. Four black vectors illustrate affective trajectories spanning different quantiles of the two-dimensional Euclidean distance. Three types of distance are defined for the transition between a starting stimulus (black dot) and ending stimulus (black arrowhead), including the two-dimensional Euclidean distance (orange), and its one-dimensional projections onto the valence (purple) and arousal (blue) axes. **(B)** Pattern similarity differences (aligned-aligned vs. aligned-misaligned) for trial pairs in which both trials had matching in-scanner choices and post-scan ratings (‘consistent’), vs. pairs containing at least one mismatch (‘inconsistent’). **(C)** Pattern similarity differences in vmPFC across quartiles of the summed Euclidean distances of the two trials in each pair. **(D)** Performance of encoding models trained on Euclidean distance and unidimensional valence and arousal distances to predict fMRI signal in vmPFC. The dotted horizontal line indicates chance-level performance. **(E)** Proportion of consistent trials across quartiles of pattern expression of Euclidean distance in vmPFC. Quartiles are used for visualization only; statistical inference was based on mixed-effects logistic regression fit to the original trial-wise binary data. The dotted horizontal line indicates chance level. Boxplots show the median and interquartile range; whiskers extend to 1.5 × the interquartile range. Black circles and error bars denote the mean and 95% confidence interval. Each point represents one participant (*n* = 18 independent participants) and points from the same participants are connected by gray lines. All numerical data are provided in S4 Data.

Given the strong relationship between affective distance and behavioral consistency (Fig 1D), we reasoned that vmPFC responses might encode affective distance separately from grid-like codes. We therefore fit multivariate encoding models predicting vmPFC responses using Euclidean distance in a two-dimensional affective space, which might correlate with decision confidence, the value of a correct decision, or the valence of stimuli (Fig 4A). Consistent with past work identifying value and decision-related variables in vmPFC [28,29,32], leave-one-participant-out cross-validation revealed encoding models using two-dimensional affective distance predicted vmPFC activity (*r* = 0.0127, 95% CI [0.0050, 0.0195], *d* = 0.7994, 38.09% of the noise ceiling, *p* = .0021) and outperformed less complex models based on valence or arousal alone (Δ*r* = 0.0097, 95% CI [0.0054, 0.0141], *d* = 1.0296, *p* = .0001; Fig 4D). This finding suggests that vmPFC activity carries information about affective distance that might be relevant for decision-making.

If representations of affective distance encoded in vmPFC were used to guide judgments made during the experiment, the predictions of encoding models should be systematically related to behavioral agreement across participants. That is, stimuli that varied more along dimensions of valence and arousal should be more consistently evaluated by participants and produce larger encoding model responses. To test this prediction, we computed trial-wise pattern expression scores by projecting the multivoxel activity onto weights from the distance encoding model. Greater expression of affective distance in vmPFC was associated with more consistent behavioral ratings (*β* = 0.0788, SE = 0.0330, *z* = 2.388, 95% CI [0.0156, 0.1430], *p* = .0169, mixed-effect logistic regression; Figs 4E and S16 Fig). This relationship remained after controlling for stimulus modality (*β* = 0.1048, SE = 0.0532, *z* = 1.969, 95% CI [0.0029, 0.2089], *p* = .0489) and did not differ between face and phrase trials (*β* = −0.0523, SE = 0.0644, *z* = −0.812, 95% CI [−0.1786, 0.0754], *p* = .4169), indicating that the association between distance-related activity and behavior did not depend on stimulus type. Moreover, hexadirectional modulation did not vary with trajectory distance (alignment × distance interaction: *β* = −0.0006, 95% CI [−0.0018, 0.0005], *d* = −0.2384, *p* = .8327; Fig 4C), indicating that the distance-related signal associated with behavior is not a simple consequence of stronger grid-like patterning. Together, these findings indicate vmPFC maintains a grid-like representation of affective space and separately encodes distance-related signals more closely related to behavioral judgments.

## Discussion

Examining brain activity as participants tracked transitions in facial expressions and language, we observed hexadirectional modulation in vmPFC consistent with grid-like coding of a two-dimensional affective space organized by valence and arousal. This representation generalized across stimulus modalities and was stable across behavioral responses. These findings link the cognitive structure described in dimensional models of emotion to grid-like coding principles previously identified in spatial and conceptual navigation.

Contemporary theories of emotion emphasize coordination among distributed brain systems, but do not specify the format in which affect is represented. Psychological constructionist accounts emphasize how systems involved in visceromotor control, interoception, and conceptualization interact with one another [35–37]. In such accounts, emotional experiences emerge from the integration of bodily states with contextual representations of the current situation to predict the outcome of possible actions, with vmPFC being one site of integration [38]. Neuroimaging and lesion studies [39] have revealed vmPFC’s involvement in affective self-report [40–42], interpretation of social cues [43,44], and prospective valuation [45,46]. The present findings extend this literature by revealing grid-like coding for affective space in vmPFC, providing a format for organizing emotion knowledge. In this framework, principles implicated in spatial and conceptual navigation may also structure how internal affective states are represented and used to guide behavior. Future work using single-unit recordings will be important to determine whether such grid-like population codes reflect periodic tuning at the level of individual neurons, as observed in rodent entorhinal grid cells during spatial navigation [27].

Our findings indicate that vmPFC exhibits grid-like coding of an affective space that is largely shared across individuals. This result is consistent with the view that emotion knowledge reflects learned regularities in affective experiences accumulated over time, rather than being constructed anew on each occasion. Responses in vmPFC were less well captured by individual post-scan ratings (S17 Fig). We do not interpret this result as evidence that vmPFC representations are insensitive to individual differences in affective experience. Individual ratings may capture meaningful participant-specific structure, but they are also more susceptible to variability in scale use and measurement noise. More densely sampled or repeated measures of each participant’s affective structure may therefore be necessary to reliably test individual-specific grid-like codes. Indeed, prior work has demonstrated meaningful variation in affective organization related to development [47] and mental health [48], including differences in emotional granularity, or the degree to which individuals differentiate emotion concepts within affective space [49]. If grid-like codes in vmPFC result from the statistical regularities of emotion transitions, then systematic variation in affect dynamics across experiences may give rise to individual differences in hexagonal modulation. Characterizing how affective space and its grid-like basis are shaped by experience will be critical for understanding the development of the affective circumplex and the mechanisms by which the brain builds structured knowledge of the world.

Differing from past studies involving learning [4,6,50], we found that the magnitude of grid-like modulation did not vary with behavioral choices, which were better predicted by distance-related signals in vmPFC. This dissociation dovetails with electrophysiological recordings in non-human primates showing temporally distinct grid-like and choice-related codes in vmPFC [24]. These findings suggest that affective information may be encoded both as abstract coordinate structure and as context-sensitive evaluative signals related to state value [51–53]. In this view, vmPFC maintains a grid-like scaffold for affective space that may support decision-relevant computations to meet the needs of the moment [54]. It is worth noting that, however, the present in-scanner behavioral task did not require participants to use an integrated representation of the valence and arousal dimensions, which might have resulted in the dissociation between grid-like coding and behavioral choice. Future studies using tasks in which judgments depend on the distance of stimuli in two-dimensional affective space can investigate whether grid-like coding is more directly related to emotional behavior when decisions require integrating information across both dimensions or if grid-like coding is less directly coupled to behavior [55]. In addition, although our findings provide evidence for the representation of two-dimensional Euclidean distance in vmPFC, the current design does not fully distinguish among alternative distance metrics that can be relevant for decision-making (e.g., Euclidean, cityblock), which will require future work that more directly dissociates these measures. More broadly, because the present findings are based on fMRI measures, they do not establish the necessity of vmPFC for organizing affective space. Future studies can use perturbation approaches and longitudinal designs to determine how grid-like coding develops and whether it is required for maintaining affective geometry or decision-making.

In sum, we show that vmPFC BOLD signal exhibits hexadirectional modulation as a function of the direction of transitions between emotion concepts. These findings suggest that the structure evident in self-report may reflect how the brain organizes emotion knowledge using mechanisms similar to those implicated in spatial and conceptual navigation. By identifying a grid-like representational format for affective space, the present work moves the study of emotion beyond the localization of function or predictive modeling toward identifying the processes that may structure affective representations in the human brain.

## Materials and methods

### Participants

We recruited 20 participants from the Atlanta, GA area. Participants were healthy adults fluent in English, with normal or corrected-to-normal vision, and no contraindications for MRI. The study was approved by the Institutional Research Board at Emory University (Protocol Number: 2025P010826), and all participants provided written informed consent prior to participation. All procedures were conducted according to the principles of the Declaration of Helsinki. Data from 18 participants (13 females; age: mean = 39.56, SD = 13.19, range = 21–60 years) were analyzed. One participant withdrew due to claustrophobia, and fMRI data from one participant were excluded due to excessive head motion (maximum framewise displacement >10 mm). Another participant did not perform the post-scan rating task and was therefore excluded from analyses using individual ratings. For the primary one-tailed paired sample comparisons, a sample size of *n* = 18 at *α* = 0.05 has 70% power to detect a true effect size of *d* = 0.53, 80% power for *d* = 0.61, and 90% power for *d* = 0.72.

### Experimental paradigm

#### Stimuli.

Photos of facial expressions were selected from the Karolinska Directed Emotional Faces database [26]. This set of photos depicts facial expressions of human models, and represents seven categories of emotions: happy, surprised, angry, afraid, sad, disgusted, and neutral. A total of 78 photos from 26 identities were used in this experiment. To construct trajectories through affective space, pairs of facial expressions from different emotion categories and the same identity were morphed into dynamic transitions using the Abrosoft FantaMorph 5 software.

Linguistic stimuli (including both multi-word expressions and single words) were selected from eight everyday settings, including ‘shopping’, ‘park’, ‘forest’, ‘school’, ‘food’, ‘animal’, ‘weather’, and ‘social’, chosen to provide distinct semantic contexts in which the same emotion categories can be conveyed. For each setting, a large candidate pool of phrases was generated using ChatGPT-4o with the following prompt ‘create a list of nouns related to [the current setting] and related to the following emotions: surprise, sadness, joy, fear, disgust, anger, neutral’. Valence, arousal, and associated emotion category for all candidate phrases were validated in a pilot study with 10 participants recruited from the Department of Psychology at Emory University. The final set of stimuli was screened such that the highest-rated category for each item matched the category assigned during generation and had a mean rank < 1.5 across pilot participants. A total of 126 phrases were used in this experiment. Pilot phrase ratings were used to guide stimulus selection and were not included in any analyses reported here.

Valence and arousal ratings used to inform the selection of the trajectories come from normative ratings of the face stimuli [56] and the averaged ratings across the pilot participants for phrases. We selected 84 face trajectories and 124 sequences of phrases for use in the scanner, aiming for balanced coverage across emotion categories, settings, and trajectory angles. For descriptive purposes, we visualized how stimulus properties varied across trajectory angles using data from the current study in S13 Fig. To formally test whether any of these factors could account for the observed hexadirectional modulation, category, context (identity or setting), trajectory distances, valence and arousal values, and behavioral choice were included as nuisance regressors in control analyses (see Materials and methods, ‘Multivariate hexadirectional modulation analysis’).

#### fMRI paradigm.

Participants completed an affective judgment task with concurrent fMRI. In this task, participants made judgments about facial expressions and sequences of phrases. The scanning session was divided into four runs, with two runs per stimulus modality. The full MRI session lasted approximately an hour, with a total task acquisition time of 36.5 min (face runs: 7.6 and 7.2 min; phrase runs: 10.9 min each). Stimuli were presented using PsychoPy v2022.2.5 [57]. The order of the two modalities (faces and phrases) and the order of the two runs within each modality were counterbalanced across participants. We performed block-wise pseudo-randomization of the order of trials within each run. For facial expressions, trajectories from the same identity were presented in a block structure; for phrases, trajectories from the same setting were grouped, and participants were cued to the current setting at the beginning of each block. Blocks lasted on average 4.73 trials (range = 1–18, SD = 5.48). Block length varied from 2.33 trials (range = 1–9, SD = 1.97) for facial expressions and 15.5 trials (range = 14–18, SD = 1.69) for phrases. This grouping was used to reduce perceptual and semantic variability across consecutive trials and to encourage participants to track affective transitions within a stable context.

In each trial of face runs, participants were presented with a 5-s morph of facial expressions, starting with one of the seven emotion categories and ending with a different category. Similarly, the stimulus in each trial of the phrase runs consisted of a starting phrase (2.5 s) followed by an ending phrase (2.5 s), forming a transition between two different emotion categories. In both the face and phrase runs, each stimulus was followed by a 1-to-5 s jittered fixation cross (mean = 2 s). After fixation, participants had 3 s to indicate whether the valence or arousal of the stimulus increased, decreased, or stayed the same. Participants made responses using their index and middle fingers on a button box in the right hand. Visual feedback was provided with a red dot to indicate ‘no change’, an ‘increase’, or a ‘decrease’. To disentangle the movement of the dot from the movement in the valence-arousal space, both ‘increase’ and ‘decrease’ were designed to be on the right of the ‘no change’. Each trial probed only one dimension (valence or arousal), with trials evenly split across dimensions. Participants were asked to track changes in both valence and arousal throughout the task, as the probed dimension was revealed at the start of the response period.

#### Post-scan self-report ratings.

After the scan, participants rated valence, arousal, and seven emotion categories on individual face expressions and phrases. The scales for valence and arousal were presented on one screen, and they ranged from ‘extremely unpleasant/calm’ to ‘extremely pleasant/activated’. The scales for the seven emotion categories (happy, surprised, angry, afraid, sad, disgusted, and neutral) were presented on another screen following the valence-arousal ratings. Category ratings ranged from ‘not at all [category name]’ to ‘extremely [category name]’. All scales were continuous, and tick marks were provided as anchor points for participants to more easily make the ratings. Participants used a mouse to click on the scale to make the ratings in a self-paced way.

These ratings were used to locate each stimulus in affective space and to compute trajectory angles for subsequent hexadirectional modulation analyses. Trajectories in both face and phrase conditions were defined geometrically as the straight line connecting the starting and ending stimuli in affective space to reflect relationship between the two stimuli, without assuming that participants mentally interpolated or represented intermediate points along the path.

### Behavioral data analysis

#### Low-dimensional organization of emotion concepts.

To assess whether emotion concepts are organized in a two-dimensional affective space in the self-report data, we performed multidimensional scaling (MDS) on the group-average ratings of emotion categories. For each stimulus, ratings on the seven emotion categories were treated as a vector, and pairwise cosine distances were computed between all stimulus pairs. The resulting dissimilarity matrix was submitted to metric MDS with dimensionality varied from 1 to 5. Model fit was evaluated using Kruskal stress [58]. The optimal dimensionality was determined based on the elbow in the scree plot of stress values.

We used Procrustes analysis [59] to align the two-dimensional MDS solution to the valence-arousal space derived from direct ratings. To assess the statistical significance of this alignment, we performed a permutation test in which the correspondence between stimuli in the valence-arousal space and the category-derived MDS space was randomly shuffled. The null distribution of Procrustes disparities was generated from 10,000 permutations, and a one-sided *p*-value was computed as the proportion of permuted Procrustes disparities less than or equal to the observed disparity.

#### Cross-modal similarity of affective structure.

To test whether the affective structure revealed in self-report is similar across stimulus modalities, we constructed separate dissimilarity matrices for face and phrase stimuli. For valence-arousal ratings, Euclidean distances were computed between stimulus pairs in the two-dimensional valence-arousal space. For emotion category ratings, cosine distances were computed between category-rating vectors. Because face and phrase stimulus sets lacked one-to-one correspondence, we used the Gromov–Wasserstein (GW) distance [60] to quantify the similarity between their structures. The Conditional Gradient algorithm [61] was used (as implemented in the Python Optimal Transport library) to estimate an optimal transport plan that aligns the two structures by minimizing the expected squared difference between their pairwise distances under the alignment. Before entering the GW computation, valence and arousal ratings were z-scored within each modality to make scales of the two spaces comparable. To test statistical significance, we performed permutation tests by disrupting the affective structure obtained from face stimuli (i.e., independently shuffling valence and arousal values or category rating vectors across stimuli), while holding the phrase-stimulus structure fixed. The null distributions of GW distances were generated from 10,000 permutations, and one-sided *p*-values were computed as the proportion of permuted GW distances that were less than or equal to the observed distance.

#### Behavioral sensitivity to affective distance during the fMRI task.

To validate that participants’ in-scanner judgments reflected the same evaluation of the stimuli captured by their post-scan ratings, we tested whether the two measures were consistent across trials. Trials were labeled as being consistent when participants’ in-scanner choice matched the difference in post-scan ratings of the judged dimension between the ending and starting stimuli. Specifically, an absolute rating difference smaller than 1 (i.e., the distance between two adjacent anchor points on the rating scale) was considered consistent with a ‘no change’ choice. If the difference was higher than 1, a choice of ‘increase’ in the scanner was considered to be consistent and vice versa if the difference was lower than −1. We then used a mixed-effects logistic regression to model the probability of a consistent choice (coded as 1) as a function of the post-scan rating change, with random intercepts and slopes for each participant.

### MRI data acquisition

Gradient-echo echo-planar imaging (EPI) data were acquired on a 3T Siemens MAGNETOM Prisma scanner with 32-channel parallel imaging located at the Facility for Education and Research in Neuroscience at Emory University. T2*-weighted functional data were acquired using a multiband multi-echo EPI sequence with the following parameters: repetition time (TR) = 1,250 ms; echo times (TEs) = 15 ms/ 37.16 ms/ 59.3 ms; flip angle = 55°; number of slices = 56 interleaved; voxel size = 2.5 mm isotropic; field of view = 210 mm; multiband acceleration factor = 4; bandwidth = 2,381 Hz/pixel. The slice angle was set to 10° relative to the anterior-posterior commissure line to reduce signal loss in basal frontal regions [62,63]. A pair of fieldmap scans with reversed phase-encoding directions (posterior–anterior and anterior-posterior) was acquired to enable correction of susceptibility-induced distortions in the functional images. Each fieldmap acquisition used the same voxel size, slice coverage and field of view as the functional scans, with TR = 7,220 ms, TE = 73 ms, and flip angle = 90°.

To facilitate normalization of EPIs to standard space, structural images were acquired using T1 MPRAGE: TR = 1,900 ms; TE = 2.26 ms; flip angle = 9°; number of slices = 192; voxel size = 1 mm isotropic; field of view = 256 mm × 256 mm; GRAPPA acceleration factor = 2; bandwidth = 199 Hz/pixel.

### MRI preprocessing

Results included in this manuscript come from preprocessing performed using *fMRIPrep* 25.0.0 [64,65] (RRID:SCR_016216), which is based on *Nipype* 1.9.2 [66,67] (RRID:SCR_002502).

#### Preprocessing of B0 inhomogeneity mappings.

One set of fieldmaps was found available within the input BIDS structure for each subject. A *B0*-nonuniformity map (or *fieldmap*) was estimated based on two (or more) EPI references with FSL’s topup [68].

#### Anatomical data preprocessing.

A total of 1 T1-weighted (T1w) images were found within the input BIDS dataset. The T1w image was corrected for intensity non-uniformity (INU) with N4BiasFieldCorrection [69], distributed with ANTs 2.5.4 [70] (RRID:SCR_004757), and used as T1w reference throughout the workflow. The T1w-reference was then skull-stripped with a *Nipype* implementation of the antsBrainExtraction.sh workflow (from ANTs), using OASIS30ANTs as target template. Brain tissue segmentation of cerebrospinal fluid (CSF), white matter (WM) and gray matter (GM) was performed on the brain-extracted T1w using FSL’s fast [71] (RRID:SCR_002823). Volume-based spatial normalization to one standard space (MNI152NLin2009cAsym) was performed through nonlinear registration with antsRegistration (ANTs 2.5.4), using brain-extracted versions of both T1w reference and the T1w template. The following template was selected for spatial normalization and accessed with *TemplateFlow* [72] (24.2.2): *ICBM 152 Nonlinear Asymmetrical template version 2009c* [73] (RRID:SCR_008796; TemplateFlow ID: MNI152NLin2009cAsym).

#### Functional data preprocessing.

For each of the 4 BOLD runs found per subject (across all tasks and sessions), the following preprocessing was performed. First, a reference volume was generated from the shortest echo of the BOLD run, using a custom methodology of *fMRIPrep*, for use in head motion correction. Head-motion parameters with respect to the BOLD reference (transformation matrices, and six corresponding rotation and translation parameters) are estimated before any spatiotemporal filtering using FSL’s mcflirt [74]. The estimated *fieldmap* was then aligned with rigid registration to the target EPI reference run. The field coefficients were mapped onto the reference EPI using the transform. The BOLD reference was then co-registered to the T1w reference using mri_coreg (FreeSurfer) followed by FSL’s flirt [75] with the boundary-based registration [76] cost-function. Co-registration was configured with six degrees of freedom.

Several confounding time-series were calculated based on the *preprocessed BOLD*: framewise displacement (FD), DVARS and three region-wise global signals. FD was computed using two formulations following Power [77] (absolute sum of relative motions) and Jenkinson [74] (relative root mean square displacement between affines). FD and DVARS are calculated for each functional run, both using their implementations in *Nipype* (following the definitions by Power and colleagues 2014 [77]). The three global signals are extracted within the CSF, the WM, and the whole-brain masks. Additionally, a set of physiological regressors were extracted to allow for component-based noise correction [78] (*CompCor*). Principal components are estimated after high-pass filtering the *preprocessed BOLD* time-series (using a discrete cosine filter with 128s cut-off) for the two *CompCor* variants: temporal (tCompCor) and anatomical (aCompCor). tCompCor components are then calculated from the top 2% variable voxels within the brain mask. For aCompCor, three probabilistic masks (CSF, WM and combined CSF + WM) are generated in anatomical space. The implementation differs from that of Behzadi and colleagues [78] in that instead of eroding the masks by 2 pixels on BOLD space, a mask of pixels that likely contain a volume fraction of GM is subtracted from the aCompCor masks. This mask is obtained by thresholding the corresponding partial volume map at 0.05, and it ensures components are not extracted from voxels containing a minimal fraction of GM. Finally, these masks are resampled into BOLD space and binarized by thresholding at 0.99 (as in the original implementation). Components are also calculated separately within the WM and CSF masks. For each CompCor decomposition, the *k* components with the largest singular values are retained, such that the retained components’ time series are sufficient to explain 50 percent of variance across the nuisance mask (CSF, WM, combined, or temporal). The remaining components are dropped from consideration. The head-motion estimates calculated in the correction step were also placed within the corresponding confounds file. The confound time series derived from head motion estimates and global signals were expanded with the inclusion of temporal derivatives and quadratic terms for each [79]. Frames that exceeded a threshold of 0.5 mm FD or 1.5 standardized DVARS were annotated as motion outliers. Additional nuisance timeseries are calculated by means of principal components analysis of the signal found within a thin band (*crown*) of voxels around the edge of the brain, as proposed by Patriat, Reynolds, and Birn (2017) [80]. All resamplings can be performed with *a single interpolation step* by composing all the pertinent transformations (i.e., head-motion transform matrices, susceptibility distortion correction when available, and co-registrations to anatomical and output spaces). Gridded (volumetric) resamplings were performed using nitransforms, configured with cubic B-spline interpolation.

Many internal operations of *fMRIPrep* use *Nilearn* 0.11.1 [81] (RRID:SCR_001362), mostly within the functional processing workflow. For more details of the pipeline, see the section corresponding to workflows in *fMRIPrep*’s documentation.

### Definition of regions of interest

Masks of the hippocampus and entorhinal cortex were obtained from the Julich-Brain Cytoarchitectonic Atlas [82]. The vmPFC mask was obtained by drawing a sphere with radius of 5 mm around the peak voxel of the hexadirectional effect reported by Constantinescu and colleagues [4] (MNI peak voxel coordinates: [6, 44, − 10]; 69 voxels). Additional vmPFC peak coordinates reported in prior studies ([6, 46, − 10] [6], [2, 28, − 20] [4], and [−8, 42, 0] [6]) were also examined using identical 5-mm-radius spherical regions of interest. For searchlight visualization within vmPFC, a larger anatomical mask including Brodmann areas 10, 11, 14, 24, 25, and 32 was defined based on a multimodal cortical parcellation of Glasser and colleagues [83]. We also conducted exploratory analyses in additional regions previously implicated in grid-like coding; the coordinates and corresponding effects are reported in S6 Table.

### General linear models

First-level general linear models were applied to preprocessed fMRI data using SPM 12 software (Wellcome Trust Centre for Neuroimaging, UK). For each participant, a separate model was estimated that included: (1) one regressor per trajectory of facial expressions or phrases, modeled as a 5 s boxcar function, (2) a regressor for button presses, modeled using a stick function, and (3) a 3-s boxcar regressor for the response period. All regressors were convolved with the canonical hemodynamic response function provided in SPM. To account for head motion, 24 nuisance regressors derived from spatial realignment (i.e., three translations, three rotations, and their first- and second-order temporal derivatives) were included, along with constant terms modeling the mean signal for each imaging session.

### Multivariate hexadirectional modulation analysis

To test whether voxel-wise activity patterns reflected 6-fold symmetry within each region of interest or searchlight, we performed a multivariate hexadirectional modulation analysis using a cross-validated procedure following Bao and colleagues [6]. The multivariate approach was used as our primary analysis because it preserves fine-grained information distributed across voxels within a region of interest [84], which can be missed by conventional univariate analysis that average across voxels. For each participant, the data were split into two halves of runs, one for estimating grid orientation (training set) and the other for testing modulation effects (test set). In the training set, we conducted a univariate analysis regressing cos(6*θ*) and sin(6*θ*) against single-trial *β*s estimated for the trajectories. The resulting regression coefficients (*β*_cos_ and *β*_sin_) were averaged across voxels within each region. The participant-specific grid orientation *φ* was then computed as


$$\begin{array}{c} \varphi =\frac{\text{1}}{\text{6}}\text{arctan}\left(\frac{\beta_{\text{sin}}}{\beta_{\text{cos}}}\right) \end{array}$$

In the test half, trials were classified as *aligned* if their trajectory angle *θ* was within ± 7.5° of *φ* (mod 60°). We computed the Pearson correlation between all pairs of response patterns for each region. Correlations were averaged across pairs of trials that were both aligned (aligned-aligned pairs, angular difference within 0° ± 15° mod 60°) and mispatched pairs (aligned-misaligned pairs with an angular difference within 30° ± 15° mod 60°). Comparisons where both trials were misaligned were excluded to avoid low-signal, high-noise pairs [6].

Trajectory angles (*θ*s) used in the analysis were obtained either from ratings of valence and arousal or coordinates in a two-dimensional MDS solution derived from pairwise cosine distances of emotion category ratings. To obtain stable MDS-derived angles, we repeated the MDS procedure with 10 random initialization seeds. One solution was selected as the reference, and all remaining solutions were aligned to it using Procrustes analysis. Trajectory angles were then computed separately for the reference and the aligned solutions, and the resulting angles were averaged across the 10 MDS solutions to yield estimates of *θ*.

To quantify the aligned-misaligned differences in pattern similarity while controlling for potential confounds, we fit a linear regression model in which pairwise pattern similarity was predicted by alignment (1 = aligned, 0 = misaligned) together with 11 nuisance regressors. These regressors captured absolute differences between the two trajectories in starting valence and arousal, ending valence and arousal, distances along the valence and arousal dimensions, and Euclidean distance in the two-dimensional space, as well as whether the pair shared the same starting emotion category, ending emotion category, behavioral choice, and stimulus context (face identity or phrase setting). Including these variables allowed us to test whether hexadirectional modulation remained after accounting for affective features, emotion category, task structure and behavior.

Because aligned trial pairs more often shared the same starting category, ending category, and face identity or phrase setting (S15 Fig), we performed a complementary matched-subsampling analysis. Within each participant and cross-validation fold, aligned and misaligned pairs were grouped according to every combination of shared starting category, shared ending category, behavioral choice, and stimulus context. For each combination, we randomly sampled without replacement an equal number of pairs from the two conditions, determined by the smaller available count. This procedure was repeated 5,000 times, producing samples of the aligned and misaligned conditions with identical numbers of pairs for each combination of the four binary variables.

To assess the specificity of the 6-fold modulation, we repeated the same procedures for alternative periodicities of 3-, 4-, 5-, 7-, and 8-fold symmetry, corresponding to angular symmetries spaced every 120° (3-fold), 90° (4-fold), 72° (5-fold), 51.4° (7-fold), and 45° (8-fold). For each periodicity *k*, *φ* was estimated using the same procedure as for the 6-fold model but applying the corresponding periodic factor. Trials were first classified as aligned if their trajectory angle *θ* was within ± (360°/(8*k*)) of *φ* (mod 360°/*k*). For each aligned trial, we computed correlations with all other trials and labeled each pair as aligned-aligned or aligned-misaligned depending on whether the angular difference between trajectories was near 0° or half of the symmetry spacing (360°/(2*k*)).

Statistical significance of group-level effects (both against 0 and for differences between regions or folds) was assessed using sign-flipping with 10,000 iterations. For each iteration, participant-specific aligned-misaligned contrast values or regression coefficients for alignment were randomly sign-inverted to generate a null distribution of group means. One-tailed *p*-values were then computed as the proportion of permuted group means that were greater than or equal to the observed group mean. To estimate uncertainty in group-level effects, we performed nonparametric bootstrap resampling (10,000 iterations) across participants, producing a distribution of group means to derive 95% confidence intervals using the percentile method [85]. Effect size was then calculated by dividing the observed group mean by the standard deviation of the bootstrap distribution, multiplied by the square root of the sample size.

### Simulation of single-trial multivoxel response patterns

To estimate the amount of variance within and between subjects in the pattern similarities, we conducted a simulation to generate single-trial patterns comprising three components: (1) a stable subject-specific pattern that was constant across trials, (2) a grid-like signal component that varied with trajectory direction, and (3) residual trial-to-trial multivoxel variation reflecting other neural and non-neural processes. Specifically, for subject *s*, trial *t*, and voxel *v*, the observed pattern of activity *Y* is:


$$\begin{array}{c} b_{s}B_{sv}+Ag_{sv}\text{cos}\left[\text{6}\left(\theta_{st}-\phi_{s}\right)\right]+\epsilon_{stv} \end{array}$$

For each participant, the pattern in the stable component *B* was derived from the empirical mean multivoxel single-trial beta pattern across trials, and the pattern in the grid-like signal component *g* was derived from the empirical voxelwise hexadirectional modulation amplitude estimated from the quadrature analysis ($\sqrt{c_{v}^{\text{2}}+s_{v}^{\text{2}}}$ where *c*_*v*_ and *s*_*v*_ are the fitted coefficients for cos(6*θ*) and sin(6*θ*), respectively). The amplitude of signals related to both spatial patterns were normalized within participants, allowing their overall contribution to the simulated data to be scaled by the parameters *b* and *A*. Subject-specific values of *b* were drawn from a normal distribution $N\left(\mu_{b},\sigma_{b}^{\text{2}}\right)$, such that *μ*_*b*_ determined the mean amplitude of the stable component across participants and *σ*_*b*_ determined its between-subject variability. The remaining trial-to-trial variation (*ϵ*) was generated from a multivariate normal distribution with the empirical cross-voxel covariance estimated after removing the fitted grid-like signal from the single-trial beta patterns.

We analyzed the simulated data using the same cross-validated procedure as the empirical data. We jointly optimized *μ*_*b*_, *σ*_*b*_, and *A* to match four empirical values including the mean raw pattern correlation, the aligned-misaligned difference in pattern correlation, the between-subject SD of the raw correlations, and the between-subject SD of the aligned-misaligned difference. For each measure, the objective was the squared difference between the simulated and empirical value. Optimal parameters were estimated using a multiobjective goal-attainment procedure implemented with MATLAB’s fgoalattain*.*

### Stability of grid orientation estimates

To assess the stability of grid orientation estimates across subsets of the data, we have performed a bootstrap analysis to compare orientation estimates across complementary split pairs. For each bootstrap iteration, we divided trials within a split into 10 contiguous temporal blocks and resampled these blocks with replacement to preserve dependence among neighboring trials. Grid orientation was then re-estimated using data from the sampled blocks. This procedure was repeated 5,000 times, producing a bootstrap distribution of the grid orientation estimates for each split and participant. Orientation stability was assessed for complementary split pairs by computing the difference between values from the two bootstrap distributions. Because the orientation estimates were defined modulo 60° for a 6-fold model, differences were wrapped to the range [−30°, 30°].

### Univariate hexadirectional modulation analysis

As a complementary test, we also assessed hexadirectional modulation using a univariate approach. For each participant, we specified a first-level general linear model including: (1) a 5-s boxcar regressor for the stimulus period, with cos(6*θ*) and sin(6*θ*) entered as parametric modulators, where *θ* was the trajectory angle through affective space, (2) a stick-function regressor for button presses, and (3) a 3-s boxcar regressor for the response period. All task regressors were convolved with the canonical hemodynamic response function. The model additionally included 24 nuisance regressors for head motion, derived from spatial realignment (i.e., three translations, three rotations, and their first- and second-order temporal derivatives), together with session-specific constants. The cos(6*θ*) and sin(6*θ*) coefficients were jointly tested using an *F* contrast to identify voxels with large linear combination of the two modulators. For group inference, each participant’s *F*-statistic map was transformed to a Z-map [86], which was then entered into a second-level one-sample *t* test. Because the first-level *F*-statistic depends on estimates of variance of the regression coefficients, biased estimation of variances in autocorrelated fMRI data can lead to inflated *F*- and transformed *Z*-statistics [4]. We therefore treated this univariate analysis as descriptive and did not use it to localize regions showing grid-like coding effect.

### Distance encoding

We built multivariate encoding models to test whether different distance measures capture variation in BOLD signal in vmPFC. Partial least squares regression (SIMPLS algorithm [87]) was used to predict multivoxel patterns of single-trial *β* estimates from distances in a cross-validated way. Encoding models were trained using data from all participants except one and tested on held-out participants (leave-one-participant-out cross-validation). For each training fold, the predictor matrix X (size 3,536 × 1) contained the distance values for each trial, and the outcome matrix Y (size 3,536 × 69) contained the corresponding single-trial *β* estimates for each voxel in vmPFC, concatenated across the 17 training participants (208 trials per participant × 69 voxels). The trained model was then used to predict responses in the held-out participant from their own distance values (test X, size 208 × 1), yielding predicted responses that were compared against the held-out participant’s observed data (test Y, size 208 × 69). Encoding performance was quantified as the Pearson correlation between the predicted and observed multivoxel single-trial responses in the test participant, computed across trials and then averaged across voxels within the region of interest to yield a single performance value per region and participant. To estimate an upper bound on model performance, the noise ceiling was calculated by correlating each held-out participant’s observed time series of brain activity with the mean time series of all other participants. One noise ceiling value was obtained for each region by averaging across held-out participants and voxels. Group-level inference for encoding model performance was conducted using the same sign-flipping and bootstrap resampling procedures as above (see ‘Multivariate hexadirectional modulation analysis’).

### Relationship between distance-related brain activity and choice behavior

To test whether the distance-related brain pattern predicts choice behavior in the scanner, we computed a pattern expression score for each trial of the held-out participant. Each participant’s observed multivoxel activity pattern (*Y*_test_) was projected onto the distance-encoding weights (*β*_d_) obtained from other participants (*Y*_test_ ⋅ *β*_d_), producing one value reflecting how strongly the response pattern on each trial expressed the distance-coding axis. These trial-wise pattern expression scores were then entered into a mixed-effects logistic regression model to predict agreement between the judgements made in the scanner and the post-scan ratings (see ‘Behavioral data analysis’ for how consistency measures were obtained). The model included random intercepts and slopes for each participant to account for individual variability. In the control analysis, we tested whether this relationship was driven by lower-level stimulus properties by including stimulus modality (face versus phrase) and its interaction with the pattern expression score as fixed effects in the model.

All analyses were performed using CanlabCore Tools (https://github.com/canlab/CanlabCore) and SPM 12 (https://www.fil.ion.ucl.ac.uk/spm/software/spm12/), as well as custom MATLAB (R2024a), python (3.9.18) and R (4.4.1) code.

## Supporting information

S1 Fig Agreement between individual and group-average post-scan ratings. **(A)** First-order agreement. Pearson correlations between individual and group-average ratings for face and phrase stimuli. **(B)** Second-order agreement. Spearman correlations between individual and group-average dissimilarity matrices derived from valence-arousal ratings (Euclidean distance) and emotion category ratings (cosine distance) for face and phrase stimuli. Black circles and error bars denote the mean and 95% confidence interval. Each point represents one subject (*n* = 18 independent subjects). All numerical data are provided in S5 Data.
(TIF)

S2 Fig Valence and arousal ratings for each participant. Each subplot shows data from one participant. Each point represents one stimulus (including both faces and phrases) and is colored according to the emotion category with the highest rating.
(TIF)

S3 Fig Multidimensional scaling (MDS) of emotion category ratings for each participant. Each subplot shows the two-dimensional MDS solution for data from one participant. Each point represents one stimulus (including both faces and phrases) and is colored according to the emotion category with the highest rating.
(TIF)

S4 Fig Low-dimensional embeddings of emotions conveyed by face and phrase stimuli. **(A)** Group-average valence-arousal ratings of face and phrase stimuli. Faces and phrases exhibited highly similar structures (Gromov–Wasserstein distance = 0.19, permutation *p* < .0001). **(B)** Multidimensional scaling solutions derived from group-average category ratings for face and phrase stimuli, aligned to the valence-arousal space in A using Procrustes analysis. Faces and phrases exhibited highly similar structures in category ratings (Gromov–Wasserstein distance = 0.01, permutation *p* < .0001). Each point represents one stimulus and is colored according to the emotion category with the highest rating.
(TIF)

S5 Fig Multidimensional scaling (MDS) of emotion category ratings. **(A)** Scree plot of Kruskal stress for MDS solutions on cosine distances between group-average ratings of emotion categories. Note the elbow at two dimensions (Kruskal stress = 0.16). **(B)** Two-dimensional MDS solution aligned to the valence-arousal space (see Fig 1C in the main text) using Procrustes analysis (disparity = 0.20, permutation *p* < .0001). Each point represents one stimulus and is colored according to the emotion category with the highest rating.
(TIF)

S6 Fig Power analysis for the observed grid-like coding effect in ventromedial prefrontal cortex. Power curve was estimated using a repeated random subsampling analysis. For each target sample size, we randomly sampled whole participants without replacement 10,000 times, performed the group-level one-sample test for each subset, and calculated power as the proportion of subsets that exhibited an effect at *a* < 0.05. This analysis revealed that the observed grid-like coding effect in the independently defined ventromedial prefrontal cortex were detected with a power exceeding 99.8% by a sample size as small as 12 participants.
(TIF)

S7 Fig Simulation of the effects of stable signal and grid-like signal strength on pattern correlations. The panels show **(A, D)** the between-subject standard deviation (SD) of the aligned correlations, **(B, E)** SD of the aligned-misaligned difference, and **(C, F)** mean aligned-misaligned difference as a function of the between-subject SD in the strength of the stable component of the simulated single-trial beta patterns. Colored lines indicate different grid-like signal amplitudes in **A–C**, and different mean amplitudes of the stable component in **D–F**. The *μ*_*b*_ was held at its fitted value in **A–C**, and *A* was held at its fitted value in **D–F**. Solid lines and points show means across 500 replicates of the simulation, shaded regions show the 2.5th–97.5th percentiles across simulations, and dashed lines indicate the corresponding empirical values. Optimization of parameters to match the empirical pattern similarity and variability measures yielded *μ*_*b*_ = 0.44, *σ*_*b*_ = 0.067, and *A* = 0.25, which produced a mean aligned-misaligned difference of 0.0072 (95% SI [0.0029, 0.0118]; empirical Δ*r* = 0.0072), an SD of the aligned correlations of 0.0294 (95% SI [0.0207, 0.0393]; empirical SD = 0.0262), and an SD of the aligned-misaligned difference of 0.0110 (95% SI [0.0073, 0.0152]; empirical SD = 0.009). At these parameter values, the grid-signal-to-noise ratio was 0.1535 on average across voxels and participants.
(TIF)

S8 Fig Whole-brain maps of signal quality. **(A)** Group temporal signal-to-noise ratio (tSNR) map. For each participant and run, voxelwise tSNR maps were created as the mean preprocessed BOLD signal divided by its standard deviation over time. The parametric map shows tSNR values averaged across runs and participants (*n* = 18 independent participants). **(B)** Mean BOLD signal across participants. For each participant, voxelwise signal intensity maps were created by averaging preprocessed BOLD timeseries across all runs. The parametric map shows the group mean of signal intensity divided by the standard deviation across participants (*n* = 18 independent participants). Note that the ventral surface of the brain (including entorhinal cortex outlined in yellow) exhibits lower tSNR and reduced mean signal intensity.
(TIF)

S9 Fig Grid-like coding effect across sphere sizes in ventromedial prefrontal cortex. Each point represents the pattern similarity difference between aligned-aligned and aligned-misaligned trial pairs for one subject (*n* = 18 independent subjects). Boxplots show the median and interquartile range; whiskers extend to 1.5× the interquartile range. Black circles and error bars denote the mean and 95% confidence interval. Across radii ranging from 3 mm (17 voxels) to 12 mm (901 voxels), the direction of effect was consistent, although its magnitude varied. We observed a “small” effect at a radius of 3 mm (*d* = 0.3826), “medium” effects at radii of 4 mm (35 voxels; *d* = 0.7687) and 5 mm (69 voxels; *d* = 0.7820), and decreasing effect sizes as the searchlight radius increases to 12 mm (*d* = 0.4116; Δ*r*_5mm_ − Δ*r*_12mm_ = 0.0042, 95% CI [0.0004, 0.0082], Δ*d* = 0.4973, *p* = .0255). Numerical data are provided in S6 Data.
(TIF)

S10 Fig Voxelwise effect sizes for searchlight hexadirectional modulation analysis within ventromedial prefrontal cortex. Searchlight maps (radius = 5 mm) reflect Cohen’s *d* values of pattern similarity differences between aligned and misaligned trial pairs across participants (*n* = 18 independent participants). These unthresholded maps are used to characterize the spatial distribution and magnitude of the hexadirectional effect within an anatomically defined mask of medial prefrontal cortex, comprising Brodmann areas 10, 11, 14, 24, 25, and 32. Warmer colors indicate greater similarity for aligned relative to misaligned trial pairs. Only voxels with positive effect sizes are displayed.
(TIF)

S11 Fig Whole-brain searchlight map of hexadirectional modulation. Parametric *t*-statistic maps show effects of hexadirectional modulation derived from an exploratory whole-brain searchlight analysis (radius = 5 mm). These maps are used to display regions showing evidence of hexadirectional modulation throughout the brain, so voxels are thresholded at *p* < .01 (one-tailed; uncorrected), with a minimum cluster extent of *k* ≥ 20 contiguous voxels for visualization. Warm colors reflect greater similarity for aligned relative to misaligned trial pairs. No voxels survived false discovery rate correction at a threshold of *q* < .05. Note that this analysis was exploratory as the sample size was chosen to test grid-like coding within independently defined, theoretically motivated regions of interest rather than to localize effects across the whole brain.
(TIF)

S12 Fig Group-level univariate hexadirectional modulation. Parametric *t*-statistic maps show the consistency across participants of the subject-level *F*-test assessing any linear combination of the cos(6*θ*) and sin(6*θ*) parametric modulators. Voxels were displayed at a false discovery rate (FDR)-corrected threshold of *q* < .05 with a minimum cluster extent of *k* = 10 contiguous voxels for visualization. Warm colors indicate stronger evidence for consistent hexadirectional modulation across participants. This univariate analysis revealed hexadirectional modulation in ventromedial prefrontal cortex (vmPFC). Multivariate analysis on a sphere (radius = 5 mm) centered on the peak vmPFC coordinate (MNI [3.5, 61.5, −18.5]; *t* = 8.8971, *Z* = 5.3596) showed that the pattern similarity difference between aligned and misaligned trial pairs was positive across participants (Δ*r* = 0.0048, 95% CI [0.0001, 0.0096], *d* = 0.4618, *p* = .0354). Patterns in the subject-level *Z* maps exhibited positive similarity to the publicly shared group-level *Z* map from Constantinescu and colleagues (2016) (mean cosine similarity = 0.1633, *t*_17_ = 5.150, *p* < 0.0001).
(TIF)

S13 Fig Distribution of stimulus properties across trajectory angles. **(A)** Proportion of highest-rated emotion categories across the four 15° bins of the trajectory angle (*θ* mod 60°) for starting and ending face and phrase stimuli. Bars show the relative frequency of categories appearing within each bin. **(B)** Distributions of valence and arousal ratings across the same four angle bins for starting and ending face and phrase stimuli. Each point represents one stimulus. **(C)** Heatmaps showing the number of trials associated with each face identity or phrase setting across the four angle bins.
(TIF)

S14 Fig Distributions of trajectory angles and distances. **(A)** Distribution of trajectory angles across four 15° bins (*θ* mod 60°), for affective space derived from valence-arousal ratings. **(B)** Distribution of trajectory distances. **(C)** Trajectory distances plotted across four 15° bins of the trajectory angle. Each point represents an individual trajectory.
(TIF)

S15 Fig Comparison of task-related factors between aligned and misaligned trial pairs in analysis on ventromedial prefrontal cortex. **(A)** Proportion of trial pairs sharing the same start category, end category, face identity or phrase setting, and behavioral choice. **(B)** Mean pairwise differences in start valence, start arousal, end valence, end arousal, trajectory distance, valence change, and arousal change. Values were calculated at the participant level after averaging within cross-validation folds. Boxplots show the median and interquartile range; whiskers extend to 1.5 × the interquartile range. Black circles and error bars denote the mean and 95% confidence interval. Each point represents one subject and points from the same subjects are connected by gray lines. All numerical data are provided in S7 Data.
(TIF)

S16 Fig vmPFC pattern expression predicts behavioral consistency. Thin blue lines show logistic regression curves fit separately for each participant. The thick black line shows the fixed-effect prediction from the mixed-effects logistic regression model. The dotted horizontal line indicates chance.
(TIF)

S17 Fig Grid-like coding effect in ventromedial prefrontal cortex using trajectory angles derived from individual ratings. Each point represents the pattern similarity difference between aligned-aligned and aligned-misaligned trial pairs for one subject (*n* = 18 independent subjects) and points from the same subjects are connected by gray lines. Boxplots show the median and interquartile range; whiskers extend to 1.5× the interquartile range. Black circles and error bars denote the mean and 95% confidence interval. We did not observe a reliable 6-fold effect in vmPFC using trajectory angles obtained from participants’ own ratings (Δ*r* = 0.0041, 95% CI [−0.0008, 0.0090], *d* = 0.4034, *p* = .0617; 6-fold vs. other folds: Δ*r* = 0.0041, 95% CI [−0.0005, 0.0086], *d* = 0.4293, *p* = .0527). Numerical data are provided in S8 Data.
(TIF)

S1 Table First-order agreement between individual and group-average ratings, and group-level reliability. (DOCX)

S2 Table Second-order agreement between individual and group-average relational structures. (DOCX)

S3 Table Stability of grid orientation estimates across complementary split pairs. (DOCX)

S4 Table Effects of hexadirectional modulation at peak coordinates in ventromedial prefrontal cortex reported in prior conceptual and olfactory navigation studies. (DOCX)

S5 Table Peak coordinates and Glasser atlas labels for clusters surviving voxelwise thresholding (*p* < .01, one-tailed; uncorrected, *k* ≥ 20 voxels) in the whole-brain searchlight analysis of hexadirectional modulation. (DOCX)

S6 Table Effects of hexadirectional modulation at peak coordinates reported in prior grid-like coding studies. (DOCX)

S1 Data Behavioral consistency across valence and arousal distances. Individual participant-level proportions of in-scanner judgments consistent with group-average post-scan ratings across quartiles of absolute distance in the judged dimension. Related to Fig 1D.
(XLSX)

S2 Data Grid-like coding effects for affective space across brain regions and periodicities. Individual participant-level pattern similarity measures. Related to Fig 2D–2F.
(XLSX)

S3 Data Grid-like coding effects for affective space after controlling for confounds and across alternative analyses. Individual participant-level pattern similarity measures from covariate-adjusted analyses, balanced subsampling, cross-modal generalization, and analyses based on a multidimensional scaling solution. Related to Fig 3B, 3C, 3E, and 3F.
(XLSX)

S4 Data Relationship between grid-like coding, distance-related signals, and behavioral consistency. Individual participant-level pattern similarity differences, encoding model performance, and proportions of consistent behavioral judgments across the corresponding analysis conditions. Related to Fig 4B–4E.
(XLSX)

S5 Data Agreement between individual and group-average post-scan ratings. Individual participant-level first-order and second-order agreement in valence-arousal and emotion category ratings of face and phrase stimuli. Related to S1 Fig.
(XLSX)

S6 Data Grid-like coding effects across sphere sizes in ventromedial prefrontal cortex. Individual participant-level pattern similarity differences across sphere radii. Related to S9 Fig.
(XLSX)

S7 Data Task-related differences between aligned and misaligned trial pairs. Individual participant-level proportions of trial pairs sharing categorical factors and mean pairwise differences in continuous factors. Related to S15 Fig.
(XLSX)

S8 Data Grid-like coding effects for affective space based on individual post-scan ratings. Individual participant-level pattern similarity differences using trajectory angles derived from participants’ own ratings. Related to S17 Fig.
(XLSX)

## Abbreviations

CSF: cerebrospinal fluid
EPI: echo-planar imaging
FD: framewise displacement
FDR: false discovery rate
GM: gray-matter
GW: Gromov–Wasserstein
INU: intensity non-uniformity
MDS: multidimensional scaling
SD: standard deviation
tSNR: temporal signal-to-noise ratio
WM: white-matter
