# Supplementary material for: Emotion knowledge is structured in a grid-like coordinate system in the human ventromedial prefrontal cortex
Source: PLoS Biol. 2026 Nov 2;24(11):e3003795. doi: 10.1371/journal.pbio.3003795 (PMC13739216; doi:10.1371/journal.pbio.3003795)
Supplement: Supplementary file 1 [file pbio.3003795.s018.docx]

| Item | Modality | Mean *r* | SD of *r* | ICC(2,k) | ICC 95% CI |
| --- | --- | --- | --- | --- | --- |
| Valence | Combined | 0.89 | 0.05 | 0.98 | 0.97, 0.98 |
| Arousal | Combined | 0.7 | 0.14 | 0.92 | 0.90, 0.94 |
| Happy | Combined | 0.88 | 0.05 | 0.97 | 0.96, 0.98 |
| Fearful | Combined | 0.75 | 0.06 | 0.95 | 0.93, 0.96 |
| Sad | Combined | 0.75 | 0.07 | 0.94 | 0.92, 0.95 |
| Surprised | Combined | 0.76 | 0.09 | 0.92 | 0.88, 0.94 |
| Angry | Combined | 0.77 | 0.06 | 0.93 | 0.90, 0.95 |
| Neutral | Combined | 0.7 | 0.21 | 0.92 | 0.90, 0.94 |
| Disgusted | Combined | 0.81 | 0.07 | 0.94 | 0.92, 0.96 |
| Valence | Face | 0.93 | 0.03 | 0.99 | 0.98, 0.99 |
| Arousal | Face | 0.76 | 0.13 | 0.95 | 0.91, 0.98 |
| Happy | Face | 0.93 | 0.04 | 0.99 | 0.98, 0.99 |
| Fearful | Face | 0.65 | 0.16 | 0.89 | 0.80, 0.95 |
| Sad | Face | 0.75 | 0.15 | 0.95 | 0.91, 0.98 |
| Surprised | Face | 0.81 | 0.15 | 0.93 | 0.87, 0.97 |
| Angry | Face | 0.81 | 0.07 | 0.91 | 0.83, 0.96 |
| Neutral | Face | 0.74 | 0.3 | 0.93 | 0.87, 0.97 |
| Disgusted | Face | 0.85 | 0.08 | 0.95 | 0.90, 0.98 |
| Valence | Phrase | 0.88 | 0.06 | 0.98 | 0.97, 0.98 |
| Arousal | Phrase | 0.68 | 0.13 | 0.92 | 0.89, 0.94 |
| Happy | Phrase | 0.86 | 0.07 | 0.97 | 0.96, 0.98 |
| Fearful | Phrase | 0.8 | 0.06 | 0.95 | 0.93, 0.96 |
| Sad | Phrase | 0.76 | 0.06 | 0.93 | 0.91, 0.95 |
| Surprised | Phrase | 0.75 | 0.08 | 0.91 | 0.87, 0.94 |
| Angry | Phrase | 0.76 | 0.08 | 0.93 | 0.90, 0.95 |
| Neutral | Phrase | 0.68 | 0.2 | 0.92 | 0.89, 0.94 |
| Disgusted | Phrase | 0.79 | 0.07 | 0.94 | 0.92, 0.96 |
