# Supplementary material for: Emotion knowledge is structured in a grid-like coordinate system in the human ventromedial prefrontal cortex
Source: PLoS Biol. 2026 Nov 2;24(11):e3003795. doi: 10.1371/journal.pbio.3003795 (PMC13739216; doi:10.1371/journal.pbio.3003795)
Supplement: Supplementary file 2 [file pbio.3003795.s019.docx]

| Rating type | Modality | Mean *r* | SD of *r* |
| --- | --- | --- | --- |
| Emotion category | Combined | 0.65 | 0.11 |
| Valence-arousal | Combined | 0.67 | 0.1 |
| Emotion category | Face | 0.72 | 0.07 |
| Valence-arousal | Face | 0.74 | 0.08 |
| Emotion category | Phrase | 0.63 | 0.11 |
| Valence-arousal | Phrase | 0.66 | 0.11 |
