# Supplementary material for: Emotion knowledge is structured in a grid-like coordinate system in the human ventromedial prefrontal cortex
Source: PLoS Biol. 2026 Nov 2;24(11):e3003795. doi: 10.1371/journal.pbio.3003795 (PMC13739216; doi:10.1371/journal.pbio.3003795)
Supplement: Supplementary file 3 [file pbio.3003795.s020.docx]

| Subject | Face 1 + Word 1 vs. Face 2 + Word 2 | | | | | |
| --- | --- | --- | --- | --- | --- | --- |
|  | 95% CI | P(\|Δφ\| ≤ 5°) | P(\|Δφ\| ≤ 10°) | P(\|Δφ\| ≤ 15°) | P(\|Δφ\| ≤ 20°) | P(\|Δφ\| ≤ 25°) |
| 1 | [-28.25°, 28.19°] | 0.19 | 0.37 | 0.55 | 0.71 | 0.86 |
| 2 | [-25.97°, 26.15°] | 0.21 | 0.44 | 0.67 | 0.84 | 0.94 |
| 3 | [-28.96°, 28.91°] | 0.08 | 0.19 | 0.33 | 0.53 | 0.76 |
| 4 | [-29.28°, 29.25°] | 0.03 | 0.08 | 0.17 | 0.36 | 0.66 |
| 7 | [3.75°, 19.14°] | 0.05 | 0.43 | 0.85 | 0.98 | 1.00 |
| 8 | [-29.47°, 29.44°] | 0.02 | 0.05 | 0.11 | 0.26 | 0.56 |
| 9 | [-28.76°, 28.77°] | 0.17 | 0.32 | 0.47 | 0.63 | 0.80 |
| 10 | [-16.02°, 19.85°] | 0.31 | 0.64 | 0.88 | 0.96 | 0.99 |
| 11 | [-28.56°, 28.36°] | 0.14 | 0.30 | 0.47 | 0.65 | 0.83 |
| 12 | [-29.36°, 29.38°] | 0.08 | 0.15 | 0.23 | 0.38 | 0.63 |
| 13 | [-29.24°, 29.25°] | 0.15 | 0.29 | 0.40 | 0.51 | 0.70 |
| 14 | [-29.34°, 29.27°] | 0.02 | 0.06 | 0.14 | 0.33 | 0.64 |
| 15 | [-28.70°, 28.87°] | 0.11 | 0.25 | 0.41 | 0.59 | 0.79 |
| 16 | [-28.74°, 28.77°] | 0.15 | 0.30 | 0.45 | 0.62 | 0.80 |
| 17 | [-28.43°, 28.58°] | 0.14 | 0.29 | 0.46 | 0.64 | 0.83 |
| 18 | [-28.38°, 28.19°] | 0.14 | 0.29 | 0.48 | 0.67 | 0.85 |
| 19 | [-27.61°, 26.25°] | 0.13 | 0.33 | 0.57 | 0.77 | 0.91 |
| 21 | [-28.24°, 28.23°] | 0.20 | 0.38 | 0.55 | 0.71 | 0.86 |
| Summary | 17/18 include 0 | Mean = 0.13 | Mean = 0.29 | Mean = 0.46 | Mean = 0.62 | Mean = 0.80 |
| Subject | Face 2 + Word 1 vs. Face 1 + Word 2 | | | | | |
|  | 95% CI | P(\|Δφ\| ≤ 5°) | P(\|Δφ\| ≤ 10°) | P(\|Δφ\| ≤ 15°) | P(\|Δφ\| ≤ 20°) | P(\|Δφ\| ≤ 25°) |
| 1 | [-29.36°, 29.33°] | 0.01 | 0.04 | 0.12 | 0.32 | 0.63 |
| 2 | [-21.20°, 22.62°] | 0.29 | 0.58 | 0.81 | 0.92 | 0.97 |
| 3 | [-28.65°, 28.56°] | 0.17 | 0.34 | 0.50 | 0.65 | 0.82 |
| 4 | [-28.43°, 28.32°] | 0.17 | 0.34 | 0.51 | 0.68 | 0.85 |
| 7 | [-9.03°, 19.05°] | 0.46 | 0.75 | 0.91 | 0.97 | 0.99 |
| 8 | [-28.59°, 28.45°] | 0.15 | 0.31 | 0.48 | 0.65 | 0.83 |
| 9 | [-28.44°, 28.35°] | 0.17 | 0.34 | 0.51 | 0.68 | 0.84 |
| 10 | [0.69°, 24.86°] | 0.08 | 0.33 | 0.69 | 0.89 | 0.97 |
| 11 | [-29.20°, 29.15°] | 0.03 | 0.12 | 0.27 | 0.46 | 0.71 |
| 12 | [-29.27°, 29.33°] | 0.10 | 0.19 | 0.28 | 0.41 | 0.66 |
| 13 | [-29.03°, 29.06°] | 0.13 | 0.26 | 0.40 | 0.56 | 0.75 |
| 14 | [-28.54°, 28.71°] | 0.13 | 0.27 | 0.43 | 0.62 | 0.81 |
| 15 | [-26.71°, 26.80°] | 0.27 | 0.50 | 0.69 | 0.82 | 0.92 |
| 16 | [-27.68°, 27.68°] | 0.24 | 0.46 | 0.63 | 0.77 | 0.89 |
| 17 | [-29.22°, 29.17°] | 0.04 | 0.11 | 0.23 | 0.43 | 0.69 |
| 18 | [-28.25°, 28.33°] | 0.12 | 0.27 | 0.47 | 0.67 | 0.85 |
| 19 | [-25.35°, 23.47°] | 0.24 | 0.55 | 0.78 | 0.89 | 0.95 |
| 21 | [-28.97°, 28.85°] | 0.08 | 0.18 | 0.33 | 0.53 | 0.77 |
| Summary | 17/18 include 0 | Mean = 0.16 | Mean = 0.33 | Mean = 0.50 | Mean = 0.66 | Mean = 0.83 |
| Subject | Face 1 + Face 2 vs. Word 1 + Word 2 | | | | | |
|  | 95% CI | P(\|Δφ\| ≤ 5°) | P(\|Δφ\| ≤ 10°) | P(\|Δφ\| ≤ 15°) | P(\|Δφ\| ≤ 20°) | P(\|Δφ\| ≤ 25°) |
| 1 | [-28.85°, 28.85°] | 0.11 | 0.24 | 0.39 | 0.58 | 0.78 |
| 2 | [-25.25°, 14.30°] | 0.32 | 0.59 | 0.78 | 0.90 | 0.96 |
| 3 | [-29.30°, 29.32°] | 0.01 | 0.06 | 0.18 | 0.38 | 0.65 |
| 4 | [-28.37°, 28.43°] | 0.11 | 0.25 | 0.44 | 0.65 | 0.84 |
| 7 | [-0.04°, 19.66°] | 0.21 | 0.65 | 0.91 | 0.97 | 0.99 |
| 8 | [-29.32°, 29.20°] | 0.00 | 0.01 | 0.07 | 0.30 | 0.65 |
| 9 | [-29.08°, 29.03°] | 0.06 | 0.16 | 0.30 | 0.50 | 0.74 |
| 10 | [-25.21°, 26.21°] | 0.27 | 0.64 | 0.81 | 0.88 | 0.94 |
| 11 | [-27.68°, 26.93°] | 0.24 | 0.46 | 0.64 | 0.78 | 0.90 |
| 12 | [-28.65°, 28.65°] | 0.11 | 0.24 | 0.40 | 0.61 | 0.81 |
| 13 | [-29.29°, 29.30°] | 0.08 | 0.18 | 0.31 | 0.48 | 0.69 |
| 14 | [-28.66°, 28.43°] | 0.07 | 0.18 | 0.35 | 0.58 | 0.81 |
| 15 | [-20.02°, 25.23°] | 0.29 | 0.56 | 0.75 | 0.88 | 0.96 |
| 16 | [-29.12°, 29.02°] | 0.01 | 0.04 | 0.13 | 0.39 | 0.72 |
| 17 | [-29.19°, 29.08°] | 0.03 | 0.09 | 0.23 | 0.45 | 0.72 |
| 18 | [-27.89°, 27.97°] | 0.20 | 0.40 | 0.58 | 0.74 | 0.88 |
| 19 | [-9.09°, 26.53°] | 0.15 | 0.39 | 0.64 | 0.83 | 0.94 |
| 21 | [-29.11°, 29.10°] | 0.05 | 0.13 | 0.27 | 0.47 | 0.72 |
| Summary | 18/18 include 0 | Mean = 0.13 | Mean = 0.29 | Mean = 0.45 | Mean = 0.63 | Mean = 0.82 |

*Note*. 95% CI indicates the 95% confidence interval of all pairwise circular differences between the two split-specific bootstrap distributions computed using the percentile method. P(|Δφ| ≤ k°) is the proportion of bootstrap pairwise differences whose absolute circular difference was within *k*° for *k* = 5, 10, 15, 20, and 25°.
