# Supplementary material for: Emotion knowledge is structured in a grid-like coordinate system in the human ventromedial prefrontal cortex
Source: PLoS Biol. 2026 Nov 2;24(11):e3003795. doi: 10.1371/journal.pbio.3003795 (PMC13739216; doi:10.1371/journal.pbio.3003795)
Supplement: Supplementary file 4 [file pbio.3003795.s021.docx]

| **Fold** | MNI [6, 44, -10]  (Constantinescu et al., 2016) | MNI [6, 46, -10]  (Bao et al., 2019) | MNI [-8, 42, 0]  (Constantinescu et al., 2016) | MNI [2, 28, -20]  (Bao et al., 2019) |
| --- | --- | --- | --- | --- |
| 6 | Δ*r* = 0.0072, 95% CI [0.0031, 0.0115], *d* = 0.7820, *p* = .0014 | Δ*r* = 0.0055, 95% CI [0.0014, 0.0096], *d* = 0.6200, *p* = .0108 | Δr = 0.0027, 95% CI [-0.0001, 0.0057], d = 0.4359, p = 0.0442 | Δ*r* = 0.0028, 95% CI [-0.0008, 0.0063], *d* = 0.3674, *p* = 0.0734 |
| 6 vs. other | Δ*r* = 0.0088, 95% CI [0.0046, 0.0133], *d* = 0.9223, *p* = .0005 | Δ*r* = 0.0064, 95% CI [0.0016, 0.0113], *d* = 0.6025, *p* = .0119 | *Δr* = 0.0036, 95% CI [0.0005, 0.0068], *d* = 0.5344, *p* = 0.0191 | *Δr* = 0.0045, 95% CI [0.0014, 0.0078], *d* = 0.6539, *p* = 0.0053 |
| 3 | Δ*r* = -0.0043, 95% CI [-0.0072, -0.0012], *d* = -0.6713, *p* = .9910 | Δ*r* = -0.0039, 95% CI [-0.0070, -0.0007], *d* = -0.5734, *p* = .9819 | Δr = -0.0048, 95% CI [-0.0074, -0.0019], d = -0.7906, p = 0.9967 | Δr = -0.0019, 95% CI [-0.0051, 0.0013], d = -0.2662, p = 0.8557 |
| 4 | Δ*r* = -0.0028, 95% CI [-0.0071, 0.0017], *d* = -0.2892, *p* = .8735 | Δ*r* = -0.0006, 95% CI [-0.0048, 0.0043], *d* = -0.0591, *p* = .5891 | Δr = -0.0002, 95% CI [-0.0030, 0.0027], d = -0.0364, p = 0.5616 | Δr = -0.0026, 95% CI [-0.0056, 0.0006], d = -0.3855, p = 0.9319 |
| 5 | Δ*r* = -0.0015, 95% CI [-0.0039, 0.0009], *d* = -0.2927, *p* = .8800 | Δ*r* = -0.0019, 95% CI [-0.0052, 0.0010], *d* = -0.2869, *p* = .8723 | Δr = -0.0028, 95% CI [-0.0056, -0.0001], d = -0.4771, p = 0.9719 | Δr = -0.0025, 95% CI [-0.0051, 0.0004], d = -0.4223, p = 0.9535 |
| 7 | Δ*r* = 0.0007, 95% CI [-0.0024, 0.0038], *d* = 0.0966, *p* = .3382 | Δ*r* = 0.0010, 95% CI [-0.0037, 0.0059], *d* = 0.0965, *p* = .3524 | Δr = -0.0012, 95% CI [-0.0042, 0.0023], d = -0.1678, p = 0.7551 | Δr = -0.0024, 95% CI [-0.0052, 0.0005], d = -0.3798, p = 0.9300 |
| 8 | Δ*r* = -0.0004, 95% CI [-0.0029, 0.0021], *d* = -0.0826, *p* = .6290 | Δ*r* = 0.0011, 95% CI [-0.0014, 0.0039], *d* = 0.1923, *p* = .2289 | Δr = 0.0044, 95% CI [0.0007, 0.0082], d = 0.5405, p = 0.0210 | Δr = 0.0009, 95% CI [-0.0012, 0.0032], d = 0.1886, p = 0.2215 |
