# Supplementary material for: Emotion knowledge is structured in a grid-like coordinate system in the human ventromedial prefrontal cortex
Source: PLoS Biol. 2026 Nov 2;24(11):e3003795. doi: 10.1371/journal.pbio.3003795 (PMC13739216; doi:10.1371/journal.pbio.3003795)
Supplement: Supplementary file 5 [file pbio.3003795.s022.docx]

| Region | Volume (cubic mm) | MNI x | MNI y | MNI z | Peak *t* | Glasser parcel | % cluster in parcel |
| --- | --- | --- | --- | --- | --- | --- | --- |
| Cblm_CrusII_L | 1608 | -28.5 | -68.5 | -42.5 | 7.03 | Cerebellum | 68 |
| Ctx_STSvp_L | 1592 | -54.5 | -32.5 | -8.5 | 6.38 | Cortex_Default_ModeB | 29 |
| Ctx_p32pr_L | 2024 | -18.5 | 13.5 | 35.5 | 5.98 | Cortex_Ventral_AttentionA | 1 |
| Ctx_TE2a_R | 1152 | 51.5 | -4.5 | -32.5 | 5.54 | Cortex_Limbic | 57 |
| Ctx_3b_L | 3424 | -20.5 | -28.5 | 55.5 | 5.35 | Cortex_SomatomotorA | 14 |
| Cau_L | 1480 | -26.5 | -10.5 | 25.5 | 5.30 | Basal_ganglia | 23 |
| Cblm_V_R | 776 | 27.5 | -42.5 | -24.5 | 5.24 | Cerebellum | 72 |
| Cblm_VI_R | 616 | 9.5 | -66.5 | -24.5 | 5.03 | Cerebellum | 61 |
| Ctx_4_R | 2584 | 43.5 | -16.5 | 29.5 | 4.90 | Cortex_SomatomotorA | 11 |
| GPi_L | 920 | -14.5 | -8.5 | -6.5 | 4.85 | Basal_ganglia | 44 |
| Ctx_11l_R | 1696 | 19.5 | 41.5 | -14.5 | 4.83 | Cortex_Fronto_ParietalB | 27 |
| Cblm_VIIIa_L | 2592 | -22.5 | -62.5 | -58.5 | 4.81 | Cerebellum | 65 |
| Cblm_I_IV_R | 816 | 5.5 | -44.5 | -16.5 | 4.80 | Cerebellum | 80 |
| Ctx_6r_R | 2768 | 41.5 | 7.5 | 27.5 | 4.80 | Cortex_Ventral_AttentionA | 17 |
| Ctx_4_R | 3696 | 13.5 | -24.5 | 65.5 | 4.79 | Cortex_SomatomotorA | 20 |
| Ctx_V1_R | 640 | 19.5 | -76.5 | 13.5 | 4.79 | Cortex_Visual_Peripheral | 46 |
| Ctx_pOFC_L | 1376 | -16.5 | 15.5 | -16.5 | 4.78 | Cortex_Limbic | 31 |
| Ctx_TGv_R | 1680 | 37.5 | 5.5 | -46.5 | 4.74 | Cortex_Limbic | 49 |
| Ctx_d32_R | 2160 | 13.5 | 39.5 | 15.5 | 4.67 | Cortex_Ventral_AttentionB | 21 |
| Ctx_6mp_L | 560 | 1.5 | -14.5 | 59.5 | 4.67 | Cortex_SomatomotorA | 34 |
| Ctx_POS2_R | 656 | 13.5 | -64.5 | 27.5 | 4.63 | Cortex_Fronto_ParietalC | 67 |
| Ctx_PCV_R | 640 | 15.5 | -50.5 | 45.5 | 4.59 | Cortex_Fronto_ParietalC | 16 |
| Ctx_V3_L | 4160 | -22.5 | -86.5 | 17.5 | 4.58 | Cortex_Visual_Central | 17 |
| Ctx_2_L | 1368 | -26.5 | -46.5 | 59.5 | 4.58 | Cortex_Dorsal_AttentionB | 25 |
| Bstem_Midbrv_R | 576 | 9.5 | -6.5 | -16.5 | 4.54 | Brainstem | 29 |
| Ctx_V4_L | 880 | -28.5 | -66.5 | -4.5 | 4.50 | Cortex_Visual_Central | 43 |
| Ctx_10v_R | 2464 | -0.5 | 43.5 | -20.5 | 4.44 | Cortex_Default_ModeA | 22 |
| Ctx_10v_R | 1416 | 9.5 | 61.5 | -2.5 | 4.43 | Cortex_Default_ModeA | 31 |
| Ctx_V3_R | 2040 | 21.5 | -90.5 | 11.5 | 4.38 | Cortex_Visual_Central | 18 |
| Ctx_PF_R | 1072 | 63.5 | -44.5 | 33.5 | 4.38 | Cortex_Ventral_AttentionA | 48 |
| Ctx_V4_R | 1200 | 35.5 | -82.5 | -10.5 | 4.36 | Cortex_Visual_Central | 44 |
| Bstem_Ponscd | 816 | 13.5 | -36.5 | -34.5 | 4.34 | Brainstem | 95 |
| Putamen_Pp_L | 808 | -24.5 | -0.5 | -12.5 | 4.31 | Basal_ganglia | 32 |
| Ctx_46_R | 936 | 29.5 | 33.5 | 35.5 | 4.31 | Cortex_Ventral_AttentionB | 23 |
| Ctx_OP1_L | 1144 | -50.5 | -22.5 | 27.5 | 4.27 | Cortex_SomatomotorB | 10 |
| Cblm_VIIb_R | 568 | 33.5 | -64.5 | -48.5 | 4.27 | Cerebellum | 75 |
| Ctx_1_L | 1376 | -56.5 | -20.5 | 51.5 | 4.26 | Cortex_SomatomotorA | 66 |
| Ctx_PeEc_L | 600 | -30.5 | -14.5 | -36.5 | 4.09 | Cortex_Limbic | 51 |
| Ctx_8BL_R | 656 | 11.5 | 43.5 | 53.5 | 4.02 | Cortex_Default_ModeB | 76 |
| Ctx_6d_L | 808 | -26.5 | -16.5 | 73.5 | 4.00 | Cortex_SomatomotorA | 49 |
| Ctx_DVT_R | 1200 | 27.5 | -66.5 | 17.5 | 3.99 | Cortex_Fronto_ParietalC | 20 |
| No label | 592 | -28.5 | 43.5 | -0.5 | 3.97 | No description | 0 |
| Ctx_a24_L | 808 | -4.5 | 37.5 | 1.5 | 3.96 | Cortex_Default_ModeA | 22 |
| Ctx_V1_L | 2120 | -12.5 | -78.5 | 5.5 | 3.93 | Cortex_Visual_Peripheral | 29 |
| Ctx_STV_R | 632 | 51.5 | -40.5 | 23.5 | 3.92 | Cortex_Temporal_Parietal | 18 |
| No label | 600 | 19.5 | 15.5 | 39.5 | 3.92 | No description | 0 |
| Cblm_VI_R | 1384 | 7.5 | -74.5 | -10.5 | 3.92 | Cerebellum | 41 |
| Caudate_Ca_R | 720 | 11.5 | 13.5 | 9.5 | 3.86 | Basal_ganglia | 74 |
| Ctx_9_46d_R | 1040 | 31.5 | 43.5 | 15.5 | 3.81 | Cortex_Ventral_AttentionB | 6 |
| GPe_R | 1360 | 19.5 | 1.5 | -2.5 | 3.76 | Basal_ganglia | 38 |
| Ctx_RI_R | 704 | 33.5 | -24.5 | 21.5 | 3.75 | Cortex_SomatomotorB | 31 |
| Ctx_OP4_L | 592 | -54.5 | -6.5 | 11.5 | 3.66 | Cortex_SomatomotorB | 45 |
| Ctx_SCEF_R | 792 | 7.5 | -4.5 | 61.5 | 3.65 | Cortex_Ventral_AttentionA | 68 |
| Ctx_1_L | 1504 | -44.5 | -20.5 | 63.5 | 3.62 | Cortex_SomatomotorA | 32 |
| Ctx_V1_L | 640 | -12.5 | -90.5 | 9.5 | 3.41 | Cortex_Visual_Peripheral | 32 |
| Ctx_7Am_L | 576 | -0.5 | -50.5 | 61.5 | 3.32 | Cortex_Dorsal_AttentionB | 74 |
| Ctx_PH_R | 632 | 53.5 | -70.5 | -10.5 | 3.12 | Cortex_Dorsal_AttentionA | 44 |
