# Supplementary material for: Emotion knowledge is structured in a grid-like coordinate system in the human ventromedial prefrontal cortex
Source: PLoS Biol. 2026 Nov 2;24(11):e3003795. doi: 10.1371/journal.pbio.3003795 (PMC13739216; doi:10.1371/journal.pbio.3003795)
Supplement: Supplementary file 6 [file pbio.3003795.s023.docx]

| Region | Effect of aligned-misaligned difference |
| --- | --- |
| TPJ, MNI [52, -42, 40] (Constantinescu et al., 2016) | Δ*r* = 0.0036, 95% CI [0.0000, 0.0075], *d* = 0.4381, *p* = .0386 |
| RSC, MNI [6, -52, 24] (Constantinescu et al., 2016) | Δ*r* = 0.0033, 95% CI [-0.0007, 0.0075], *d* = 0.3765, *p* = .0700 |
| PCC, MNI [0, -32, 28] (Constantinescu et al., 2016) | Δ*r* = 0.0012, 95% CI [-0.0030, 0.0048], *d* = 0.1396, *p* = .3016 |
| LPC, MNI [30, -62, 28] (Constantinescu et al., 2016) | Δ*r* = -0.0005, 95% CI [-0.0052, 0.0042], *d* = -0.0449, *p* = .5771 |
| mPFC, MNI [-3, 63, 15] (Doeller et al., 2010) | Δ*r* = 0.0012, 95% CI [-0.0045, 0.0066], *d* = 0.0998, *p* = .3435 |
| PPC, MNI [-18, -54, 45] (Doeller et al., 2010) | Δ*r* = 0.0018, 95% CI [-0.0010, 0.0047], *d* = 0.2847, *p* = .1287 |
| Left LTC, MNI [-54, 9, -30] (Doeller et al., 2010) | Δ*r* = -0.0003, 95% CI [-0.0052, 0.0048], *d* = -0.0232, *p* = .5400 |
| Right LTC, MNI [42, 15, -36] (Doeller et al., 2010) | Δ*r* = -0.0001, 95% CI [-0.0031, 0.0035], *d* = -0.0103, *p* = .5149 |

*Note.* TPJ = temporoparietal junction. RSC = retrosplenial cortex; PPC = posterior parietal cortex; LTC = lateral temporal cortex; mPFC = medial prefrontal cortex; PCC = posterior cingulate cortex; LPC = lateral parietal cortex; Reported *p* values are uncorrected.
